# pH-sensor GPR68 plays a role in how dietary fibre lowers blood pressure in a preclinical model of hypertension

**DOI:** 10.1042/CS20243009

**Published:** 2025-10-28

**Authors:** Evany Dinakis, Chudan Xu, Rikeish R. Muralitharan, Hamdi Jama, Liang Xie, Kwan Charmaine Leung, Katrina M. Mirabito Colafella, Zoe McArdle, Ekaterina Salimova, Leticia Camargo Tavares, Matthew Snelson, Chad Johnson, Tracey Gaspari, Charles R. Mackay, Joanne A. O’Donnell, Francine Z. Marques

**Affiliations:** 1 Hypertension Research Laboratory, Department of Pharmacology, Biomedical Discovery Institute, Faculty of Medicine, Nursing and Health Sciences, Monash University, Clayton, Australia; 2 Victorian Heart Institute, Monash University, Melbourne, Australia; 3 Department of Microbiology, Monash Biomedicine Discovery Institute, Monash University, Melbourne, Australia; 4 Precision Medicine Translational Research Programme, Department of Obstetrics & Gynaecology, Yong Loo Lin School of Medicine, National University of Singapore, Singapore; 5 Cardiovascular Disease Program, Department of Physiology, Monash Biomedicine Discovery Institute, Monash University, Melbourne, Australia; 6 Monash Bioimaging Facility, Monash University, Melbourne, Australia; 7 Bioimaging Platform, La Trobe University, Melbourne, Australia; 8 IRAP Pharmacology Group, Department of Pharmacology, Monash Biomedicine Discovery Institute, Monash University, Melbourne, Australia; 9 School of Pharmaceutical Sciences, Shandong Analysis and Test Center, Qilu University of Technology (Shandong Academy of Sciences), Jinan 250014, China; 10 Baker Heart and Diabetes Institute, Melbourne, Australia

**Keywords:** diet, G protein-coupled receptor, hypertension, immune cells, microbiome, pH, blood pressure

## Abstract

Dietary fibre lowers blood pressure (BP) via short-chain fatty acids, acidic metabolites released from fibre fermentation by bacteria in the large intestine. This acidic microenvironment may activate the pH-sensing receptor GPR68, primarily expressed in immune cells. Here, we aimed to investigate whether GPR68 confers the BP-lowering effects of a high-fibre diet in hypertension by regulating inflammatory responses. Baseline BP parameters were measured using telemetry in C57BL/6J wildtype (WT) and GPR68-deficient (*Gpr68^−/−^*) male and female mice. Moreover, male mice were fed a control or high-fibre diet following minipump implantation with saline or angiotensin II (Ang II), where BP was measured weekly by tail-cuff. Cardiac ultrasounds, histological, flow cytometric and gut microbiome (16S) analyses were performed. No BP differences were detected in untreated male and female mice, irrespective of genotype. Similarly to WT mice, *Gpr68^−/−^* male mice were susceptible to Ang II-induced hypertension. High-fibre-fed WT mice exhibited blunted elevations in BP and improved cardiac collagen deposition and aortic elastin content compared with control-fed WT mice. These were not observed in high-fibre-fed *Gpr68^−/−^* mice. A high-fibre diet decreased pro-inflammatory renal and aortic immune cell counts independently of GPR68. Dietary fibre, rather than GPR68 or Ang II, was the primary factor influencing differences in the gut microbiota. This study provides novel insight into how the pH-sensing receptor GPR68 may be implicated in the protective effects of a high-fibre diet. However, these effects are likely immune-independent.

## Introduction

Lifestyle interventions, including those involving diet, are first-line therapy for hypertension treatment and prevention of cardiovascular disease (CVD) [1]. Notably, higher fibre consumption is associated with a 15–30% reduction in CVD incidence and mortality [2]. Dietary fibres are particularly relevant as they evade absorption and digestion in the upper gastrointestinal (GI) tract [3,4]. The microbiota in the large intestine primarily ferments prebiotic fibre, such as soluble fibre and resistant starches [5]. This fermentation produces bioactive metabolites known as short-chain fatty acids (SCFAs), predominantly acetate, butyrate and propionate [6]. Using pre-clinical models of hypertension, we and others demonstrated that SCFAs underpin the cardioprotective effects and associated modulation of the gut microbiota conferred by dietary fibre [7–10]. Direct administration of acetate and butyrate successfully lowered blood pressure (BP) by 35% and 20%, respectively [7,10]. More recently, these findings were clinically validated, with colonic delivery of acetate and butyrate-enriched fibre lowering 24-hour systolic BP in untreated hypertensive participants and fostering the expansion of faecal microbes that produce SCFAs [11]. The immune system and low-grade inflammation are recognised as key mechanisms involved in the pathogenesis and progression of hypertension and, more widely, CVD [12–15]. Importantly, the digestive system contains a relatively high proportion of lymphocytes, which make up approximately 70% of the immune cells in the GI tract, with SCFAs playing an essential role in immune function and regulation [16–18].

How SCFAs interact with the cardiovascular system to lower BP remains to be elucidated. A growing body of evidence supports the role of G protein-coupled receptors (GPCRs) that directly sense SCFAs, such as GPR41, GPR43 and GPR109A [7,19–21]. However, these do not explain all cardioprotective mechanisms observed via high-fibre or SCFA interventions. A new hypothesis is based on the acidic nature of SCFAs, as their accumulation following fibre fermentation is accompanied by decreases in colonic luminal pH [22–24]. pH regulation is essential for normal biological physiology, cell metabolism and function [25]. Under homeostatic conditions, the physiological pH of humans’ circulation and tissues is tightly maintained between 7.35 and 7.45 [26]. However, certain organ systems, such as the large intestinal tract, require a constant acidic pH to maintain normal barrier function and protection [27]. Shifts in acidic pH, such as the accumulation of SCFAs following fibre fermentation, lower large intestinal luminal and interstitial fluid pH significantly compared with a standardised diet [28]. Physiological shifts in acidic pH may be sensed by a family of proton-sensing GPCRs, such as GPR68 [29]. These GPCRs are highly sensitive to mild, physiological shifts in acidic pH [30]. Although pH-sensing GPCRs slightly differ in their maximal activation thresholds, GPR68 remains applicable to SCFA-induced reductions in colonic pH as its maximal activation lies at ~pH 6.8 [29]. GPR68 is expressed in a number of different cell types, including epithelial cells of the intestine and renal tubules, vascular smooth muscle cells and fibroblasts [31–33]. However, it is particularly enriched in immune cells [32] and is involved in a suite of immune responses that are applicable in hypertension [32,34,35]. For example, GPR68 is expressed in CD4^+^ and CD8^+^ T cells, myeloid-derived cells, natural killer (NK) and dendritic cells [32,36–38]. Furthermore, GPR68 mediates immune cell activation by suppressing macrophage and T-cell infiltration and the subsequent production of pro-inflammatory cytokines in a setting of nephropathy [35]. Both of these are known contributors to hypertension and associated end-organ damage [39–41].

Therefore, in this study, our primary aim was to determine whether GPR68 confers the BP protective effects of dietary fibre in a pre-clinical model of hypertension. Our secondary aims were to determine changes in hypertension-associated end-organ damage (e.g. fibrosis), immune cells and the gut microbiota. We hypothesised that acidification of colonic pH, following fibre fermentation and subsequent SCFA production, activates GPR68 signalling and reduces the accumulation of pro-inflammatory immune cells in the kidneys and aorta.

## New and noteworthy

Dietary fibre lowers BP via gut microbial metabolites that lower large intestinal pH. This could activate signalling via pH-sensing mechanisms. We demonstrate that pH-sensing receptor GPR68 may offer a new approach for leveraging the BP-lowering effects of dietary fibre in hypertension, likely through immune-independent pathways. Additionally, we show that dietary fibre alters large intestinal physiology and the gut microbiota in hypertensive mice independently of GPR68.

## Methods

### Experimental studies

All animal care and experimental procedures conducted in this study were approved by the Animal Ethics Committee of Monash University (AEC 27929 & 41271). WT C57BL/6 J mice were obtained from the Monash Animal Research Platform, Monash University. Whole-body single-knockout *Gpr68* mice on a C57BL/6J background were established by C.R.M. at Monash University. Mice were generated using a CRISPR/Cas9-based protocol at the Monash Genome Modification Platform at Monash University. Briefly, the UCSC Genome Browser was used to identify guide RNA target sites flanking the *Gpr68* gene. The following guide RNA was used: 361 base pairs upstream of the ATG region of *Gpr68* (5′ CCCCCTGGTACAAGCAATGG 3′) and 340 base pairs downstream of the STOP codon of *Gpr68* (5′ GCTTGGCAGGTGACCACGCA 3′) to knock out the *Gpr68* gene. CRISPR RNAs (crRNAs, IDT) were annealed with trans-activating crRNA (tracrRNA) to form a functional crRNA:tracrRNA guide RNA duplex. Cas9 nuclease (IDT, # 1081058) was incubated with the guide RNAs to form a ribonucleoprotein complex. Cas9 nuclease (30 ng/μl) and the crRNA:tracrRNA guide RNA duplexes (30 ng/μl) were microinjected into the pronucleus/cytoplasm of the zygotes at the pronuclei stage. Injected zygotes were transferred into the uterus of pseudo-pregnant F1 females. Genome-edited F1 *Gpr68^−/−^* mice were bred with C57BL/6 mice for two rounds to dilute off-target effects. *Gpr68^−/−^* littermates were generated by breeding heterozygote (*Gpr68^+/−^*) parents. Mice were maintained as a homozygous or heterozygous breeding colony and routinely genotyped. All mice were maintained under specific pathogen-free and controlled environmental conditions. A total of 100 male and female mice were used in this project (Supplementary Table S1).

### Blood pressure measurements

Baseline BP was monitored via radiotelemetry in eight-to-ten-week-old untreated male and female littermate WT and *Gpr68^−/−^* mice. Briefly, mice were anaesthetised using 1–2% isoflurane inhalation for implantation of a radiotelemetry probe (TA11PA-C10, Data Sciences International) into the carotid artery, and the transmitter body was placed subcutaneously along the right flank. Following a ten-day recovery period, parameters including systolic BP, diastolic BP, mean arterial pressure (MAP), heart rate and activity were measured by sampling 10 seconds every 10 minutes using the Ponemah software (Data Sciences International) for three days. Activity was measured as locomotor activity detected in arbitrary units, where inactivity was defined as 0, and >0 represents the active state. All recordings from each 24-hour period were averaged. Additionally, recordings were averaged separately for the day (7 AM to 7 PM) and night (7 PM to 7 AM) cycles to compare differences. Day–night cycles were controlled by automated lighting in the mice room.

For mice subjected to minipump surgery, systolic BP was measured using the CODA High Throughput Non-invasive Tail-cuff system (Kent Scientific Corporation). Measurements and analysis were performed according to the manufacturer’s protocol. Briefly, mice underwent three consecutive days of acclimatisation, followed by baseline BP measurements at least one day before osmotic minipump surgeries. Following minipump implantation, animals recovered for at least five days before subsequent weekly BP was recorded for four consecutive weeks. Five acclimatisation cycles and 15 measurement cycles were recorded per mouse. At least eight acceptable measurements were used to calculate average systolic BP.

### Angiotensin II model of hypertension

Six-to-eight-week-old male WT and *Gpr68^−/−^* mice underwent subcutaneous osmotic minipump (ALZET 2004) surgery under isoflurane anaesthesia to receive either 0.9% saline (sham) or a slow-pressor dose of angiotensin II (Ang II, 0.75 mg/kg body weight/day; Auspep) in the lower flank region. Osmotic minipumps were prepared and primed for 40 hours before implantation at 37°C. WT and littermate *Gpr68^−/−^* mice were randomised into either group using an Excel randomisation tool.

### Dietary interventions

All mice were allowed to feed ad libitum during the experimental timeline. The two dietary interventions used were a control diet (AIN93G) and a nutrient-matched diet high in resistant starches (referred to as high-fibre, SF11-025), both obtained from Speciality Feeds (see Supplementary Table S2 for nutritional information). At the initiation of the experimental timeline (i.e. before acclimatisation for BP measurements), mice were placed on the control diet. Immediately after surgery, mice were either maintained on the control diet or switched to a high-fibre diet for the four-week protocol. Food was replenished as necessitated. Dietary intervention randomisation was performed using an Excel randomisation tool.

### Cardiac ultrasounds

Up to five days before euthanasia, echocardiography to image the left ventricle using a Vevo 2100 Imaging System was performed. Mice were anaesthetised using 1–2% isoflurane inhalation, and parasternal long-axis and short-axis (B-mode) and M-mode images were acquired by an experienced technician (H.A.J.) at the Monash Biomedical Imaging Centre. Two to three images were acquired per parameter. Two independent researchers validated the analysed images (E.D., H.A.J.). Image acquisition, analysis and validation were all performed blindly.

### Endpoint tissue collection

At the endpoint, mice were killed by CO_2_ asphyxiation. Blood was collected via cardiac puncture and perfusion with Dulbecco’s phosphate buffered saline (dPBS, 1X, D8537, Sigma-Aldrich). Tissue samples were excised and either processed for histological examination or snap-frozen in liquid nitrogen and stored at −80°C until further analysis. Specifically, the thoracic aorta, one kidney and the spleen were placed in dPBS and kept on ice until processing for flow cytometric analyses.

### Histological analyses

Following dissection, a 2–3-mm section of the thoracic aorta was excised, the heart was transversely sectioned, and one kidney was sectioned along the coronal plane. The basal portion of the heart and one-half of the sectioned kidney were used for histology. The colon was isolated from the caecum and gently flushed with dPBS to encourage removal of contents. The colon was then longitudinally incised, retracted and swiss-rolled to preserve its structural integrity. Prepared tissue sections were stored in 10% neutral buffered formalin for 24–48 hours prior to paraffin embedding for histopathological analyses. Paraffin-embedded heart, kidney and aorta tissue blocks were sectioned at 4 μm and 10 μm for colon tissue blocks by the Monash Histology Platform. Masson’s trichrome staining was performed on heart, kidney and colon sections to quantify collagen deposition. Alcian blue/periodic acid–Schiff staining was also performed on colon samples to quantify goblet cells and muscularis propria layer thickness. Verhoeff–Van Gieson staining was performed on aorta samples for elastin quantification. Whole tissue sections were scanned at 40× magnification with the Scanscope AT Turbo (Aperio). Total collagen deposition measurements were quantified in heart and kidney samples using a colour-thresholding macro in ImageJ. Heart samples were further analysed for perivascular and interstitial collagen deposition using two separate colour-thresholding macros in ImageJ. Specifically, five vessels of medium to large size were imaged per heart sample at 20× magnification for perivascular collagen deposition analysis, and 10× magnification in five different fields of view for interstitial collagen deposition analysis using ImageScope software (Aperio, v12.4.0.5043). Each colon section was captured at 10× magnification in three different fields of view for quantification of collagen deposition, muscularis propria layer thickness and goblet cell counts. All analyses were performed manually using FIJI ImageJ software. Briefly, the thickness of collagen deposition within the submucosa was quantified over an average length of 250 μm within each field of view, and an overall average of the three fields of view was taken per tissue section and reported as collagen deposition thickness (μm). The thickness of the muscularis propria layer was quantified over an average area of 35 mm^2^ and an average length of 250 μm within each field of view, and an overall average of the three fields of view was taken per tissue section and reported as muscularis propria thickness (mm). Goblet cell counts were quantified over an average area of 70 mm^2^ within each field of view, and an overall average of the three fields of view was taken per tissue section and reported as the number of goblet cells (per mm^2^ area). Whole tissue sections of the aorta were captured at 10× magnification and analysed manually using FIJI ImageJ software. Briefly, each image was colour deconvoluted to distinguish elastin staining. A set threshold was then applied to quantify the inner and outer layers of elastin and reported as total medial elastin (%). Image acquisition and analysis were performed in a blinded manner.

### Isolation of mouse immune cells for flow cytometric analyses

Mouse splenic cell suspensions were prepared through mechanical disruption and filtration through a 70-μm mesh strainer. At this time, splenic cells and 100 μl of blood were subjected to red blood cell lysis for 5 minutes (Biomedicine Learning and Teaching Building & Media Services Facility), followed by a cold dPBS wash and cell isolation spin at 1200 rmp at 4°C for 10 minutes. At this point, splenic cell suspensions were ready for staining. Blood samples were subjected to a second red cell lysis buffer treatment, followed by another cold dPBS wash and cell isolation spin at 1200 rpm at 4°C for 10 minutes. At this point, peripheral blood cell suspensions were ready for staining. For mouse renal and aortic cell suspensions, one kidney per mouse and the thoracic aorta were mechanically disrupted and enzymatically digested in 1.5 ml of RPMI 1640 (Biomedicine Learning and Teaching Building & Media Services Facility), 1.8 mg/ml Collagenase type I-S (C1639, Sigma-Aldrich), 0.156 mg/ml Collagenase type XI (C7657, Sigma-Aldrich) and 0.030 mg/ml Hyaluronidase type IV-S (H3884, Sigma-Aldrich) for 60 minutes on an orbital shaker at 180 rpm at 37°C. Following digestion, cell suspensions were filtered through a 70-μm mesh strainer and centrifuged at 1200 rpm at 4°C for 10 minutes. At this point, aortic cell suspensions were ready for staining. Renal cell suspensions were subjected to a 40%/60% Percoll (GE17-0891-01, Sigma-Aldrich) gradient separation spin at 2700 rpm at 18°C for 25 minutes with no deceleration. The mononuclear cells at the interface level were collected, washed in cold dPBS, and underwent a cell isolation spin at 1200 rpm at 4°C for 10 minutes. At this point, all cell suspensions were ready for staining.

### Flow cytometry

Cells were resuspended in FACS buffer [dPBS containing 2% foetal bovine serum (12007C, Sigma-Aldrich) and 2 mM ethylenediaminetetraacetic acid (Monash Biomedicine Learning and Teaching Building & Media Services Facility)]. Splenic cell counts were performed manually using a haemocytometer and Trypan blue (0.4%, T10282, Invitrogen). In total, 10^6^ splenocytes were used for staining; renal, aortic and blood cell counts were determined using flow cytometry counting beads (C36950, Invitrogen). Cell viability was determined using a fixable viability stain (LIVE/DEAD™ Fixable Blue Dead Cell Stain Kit, for UV excitation, L34962, Invitrogen) for 30 minutes at 4°C in the dark. Single-cell suspensions were then stained for surface antigen receptors for 30 minutes at 4°C in the dark using the antibodies listed in Supplementary Tables S3–S5. Cells were then fixed and permeabilised using the eBioscience^TM^ Foxp3/Transcription Factor Staining Buffer Set (00–5523-00, Invitrogen), followed by intracellular staining with Foxp3 for 15 minutes at room temperature. Samples were acquired on a five-laser BD LSRFortessa X-20 flow cytometer using BD FACSDiva software (BD Biosciences). Analyses were performed using FlowJo v10.10.0. Gating strategies are provided in Supplementary Figures S1–S3. Major immune cell populations in the blood, spleen and kidney were defined as follows: overall immune cells (CD45^+^), macrophages (CD45^+^CD11b^+^F4/80^+^), inflammatory monocytes (CD45^+^CD11b^+^Ly6C^hi^), neutrophils (CD45^+^CD11b^+^Ly6G^+^Ly6C^int^), dendritic cells (CD45^+^CD11c^+^MHCII^+^), B cells (CD45^+^B220^+^MHCII^+^), γδ T cells (CD45^+^TCRγδ^+^), CD4^+^T cells (CD45^+^TCRβ^+^CD4^+^), CD8^+^T cells (CD45^+^TCRβ^+^CD8^+^), T_reg_ cells (CD45^+^TCRβ^+^CD4^+^Foxp3^+^) and NK cells (CD45^+^NK1.1^+^). Dendritic cells were further categorised as conventional dendritic cells type 1 (CD45^+^CD11c^+^MHCII^+^CD11b^-^), and conventional dendritic cells type 2 (CD45^+^CD11c^+^MHCII^+^CD11b^+^) in non-lymphoid tissues [42]. Major immune cell populations in the aorta were defined as follows: macrophages (CD45^+^CD11b^+^F4/80^+^), inflammatory monocytes (CD45^+^CD11b^+^Ly6C^hi^), neutrophils (CD45^+^CD11b^+^Ly6G^+^Ly6C^int^), B cells (CD45^+^B220^+^), CD4^+^T cells (CD45^+^CD4^+^), CD8^+^T cells (CD45^+^CD8^+^) and NK cells (CD45^+^NK1.1^+^).

### Caecal DNA extraction, library preparation and 16S sequencing

Caecal DNA (0.1 g) was extracted using the DNeasy PowerSoil DNA isolation kit (Qiagen) according to the manufacturer’s protocol. DNA libraries were prepared by polymerase chain reaction (PCR) amplification of the V4 region of bacterial 16S ribosomal RNA (rRNA) as previously described [43] using 20 ng of caecal DNA, 515F (5′-GTGCCAGCMGCCGCGGTAA-3′) and 806R (5′-GGACTACHVGGGTWTCTAAT-3′) primers and Platinum Hot Start PCR master mix in a Thermal Cycler (BioRad). DNA library concentrations were quantified using a Qubit (ThermoFisher Scientific). Two hundred and forty nanogram of the library was sequenced in an Illumina MiSeq sequencer (300 bp paired-end reads).

### 16S ribosomal RNA bioinformatics analyses

Data were processed using nf-core/ampliseq version 2.7.1 [44] of the nf-core collection of workflows [45], utilising reproducible software environments from the Bioconda [46] and Biocontainers [47] projects. Data quality was evaluated with FastQC and summarised with MultiQC [48]. Sequences were processed independently for each sample using DADA2 [49] to eliminate PhiX contamination, trim reads (forward reads at 220 bp and reverse reads at 210 bp, reads shorter than this were discarded), discard reads with >2 expected errors, correct errors, merge read pairs and remove PCR chimeras; ultimately, 535 amplicon sequencing variants (ASVs) were obtained across all samples. An average of 65.5% reads per sample were retained. The ASV count table contained 3,294,075 counts in total, a minimum of 10,961 and a maximum of 102,428 counts per sample, with an average of 45,751 counts. Taxonomic classification was performed by DADA2 and the database ‘Silva 138.1 prokaryotic SSU’ [50]. ASV sequences, abundance and DADA2 taxonomic assignments were loaded into QIIME2 [51]. Of 535 ASVs, one was removed due to taxonomic string contamination; therefore, 534 ASVs passed. All downstream analyses were performed on rarefied samples using MicrobiomeAnalyst 2.0 [52]. Briefly, features with a minimum of four counts occurring at a prevalence of 20% in samples were included, and a low variance removal filter was applied at 10% based on the inter-quartile range. Data were scaled using the Total Sum Scaling normalisation method to account for technical bias associated with varying sequencing depths in different libraries [53]. Abundance profiling of bacterial families, feature-level measurements of α-diversity and β-diversity metrics, including unweighted and weighted UniFrac metrics shown as Principal Coordinate Analysis (PCoA) plots, were performed on rarefied samples. Species-level multiple linear regression analysis with covariate adjustment for genotype was performed using Microbiome Multivariate Association with Linear Models with a false discovery rate (FDR) q-value of 0.05 used as threshold [54].

### Statistical analyses

GraphPad Prism v9.3.1 was used for all statistical analyses and graphing. Normal distribution of data was assessed using Shapiro–Wilk’s normality test. To identify outliers within datasets, the ROUT method was applied with Q set at 1%. To ensure data integrity, values that did not pass the outlier detection criteria were excluded from the final analysis and are not represented in the figures shown. For normally distributed data, one-way or two-way analysis of variance (2-way ANOVA with Benjamini and Hochberg’s FDR adjustment for multiple comparisons) was performed to compare groups. For non-normally distributed data, the non-parametric Kruskal–Wallis test was applied. Repetitive measures analyses were used for weekly tail-cuff BP only. Pairwise permutational multivariate analysis of variance (PERMANOVA) analysis was performed on unweighted and weighted UniFrac metrics. Unless otherwise stated, all values are represented as mean ± SEM, and *P*<0.05 (or FDR adjusted q<0.05) was considered statistically significant.

## Results

### GPR68 deficiency does not influence differences in cardiovascular phenotypes

**Figure 1:**
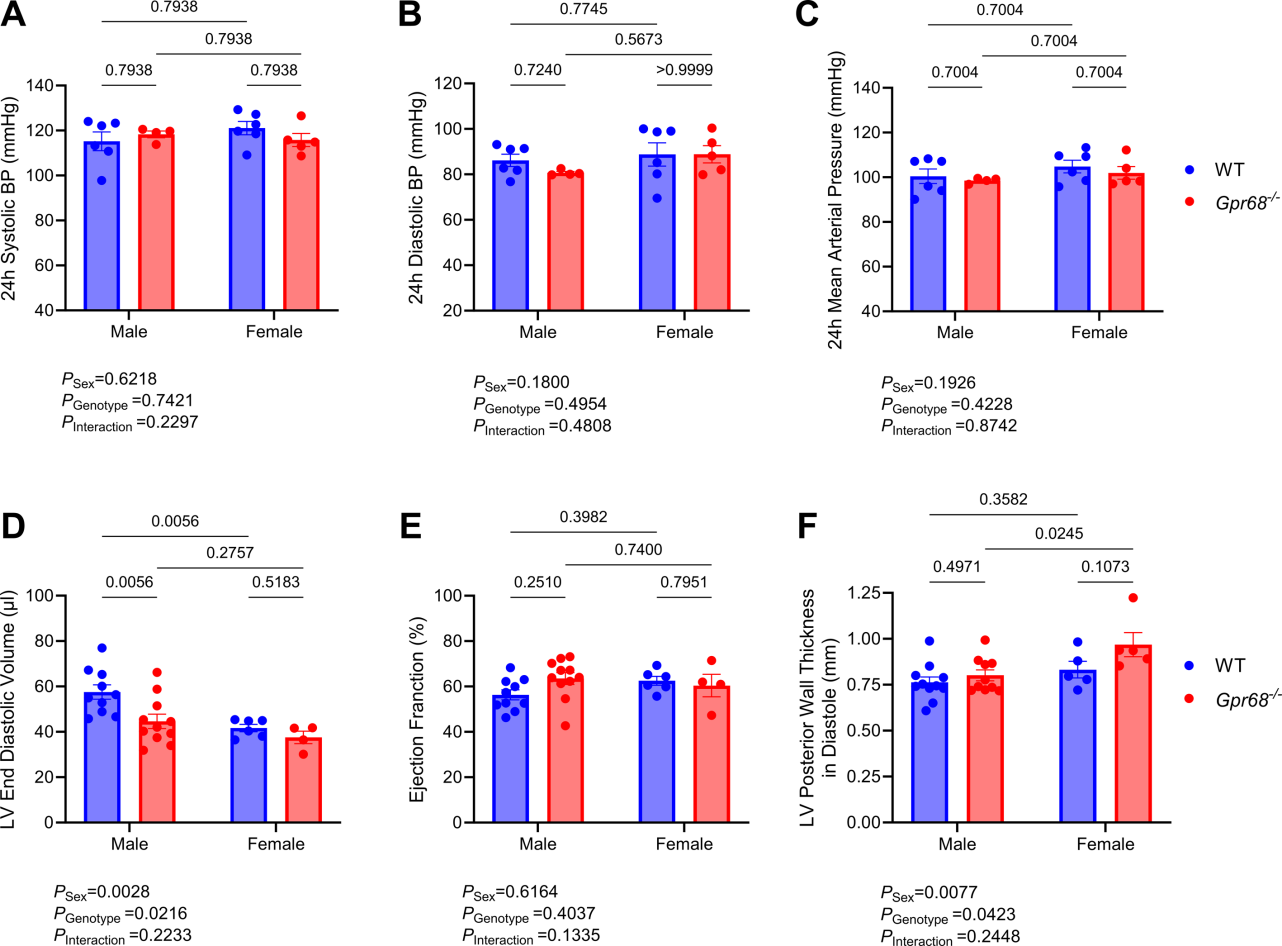
Characterisation of GPR68 deficiency in cardiovascular phenotypes of male and female wildtype (WT) and *Gpr68* ^−/−^ mice. 24-hour blood pressure (BP) measured by telemetry in naïve eight-to-ten-week-old mice including (**A**) systolic, (**B**) diastolic and (**C**) mean arterial pressure. Cardiac ultrasound data, including (**D**) left ventricular (LV) end diastolic volume, (**E**) ejection fraction and (**F**) LV posterior wall thickness in diastole. Each data point represents an individual sample. Two-way ANOVA with Benjamini and Hochberg’s false discovery rate adjustment for multiple comparisons, showing q-value. Data presented as mean ± SEM. *n*=4–11/group.

**Figure 2:**
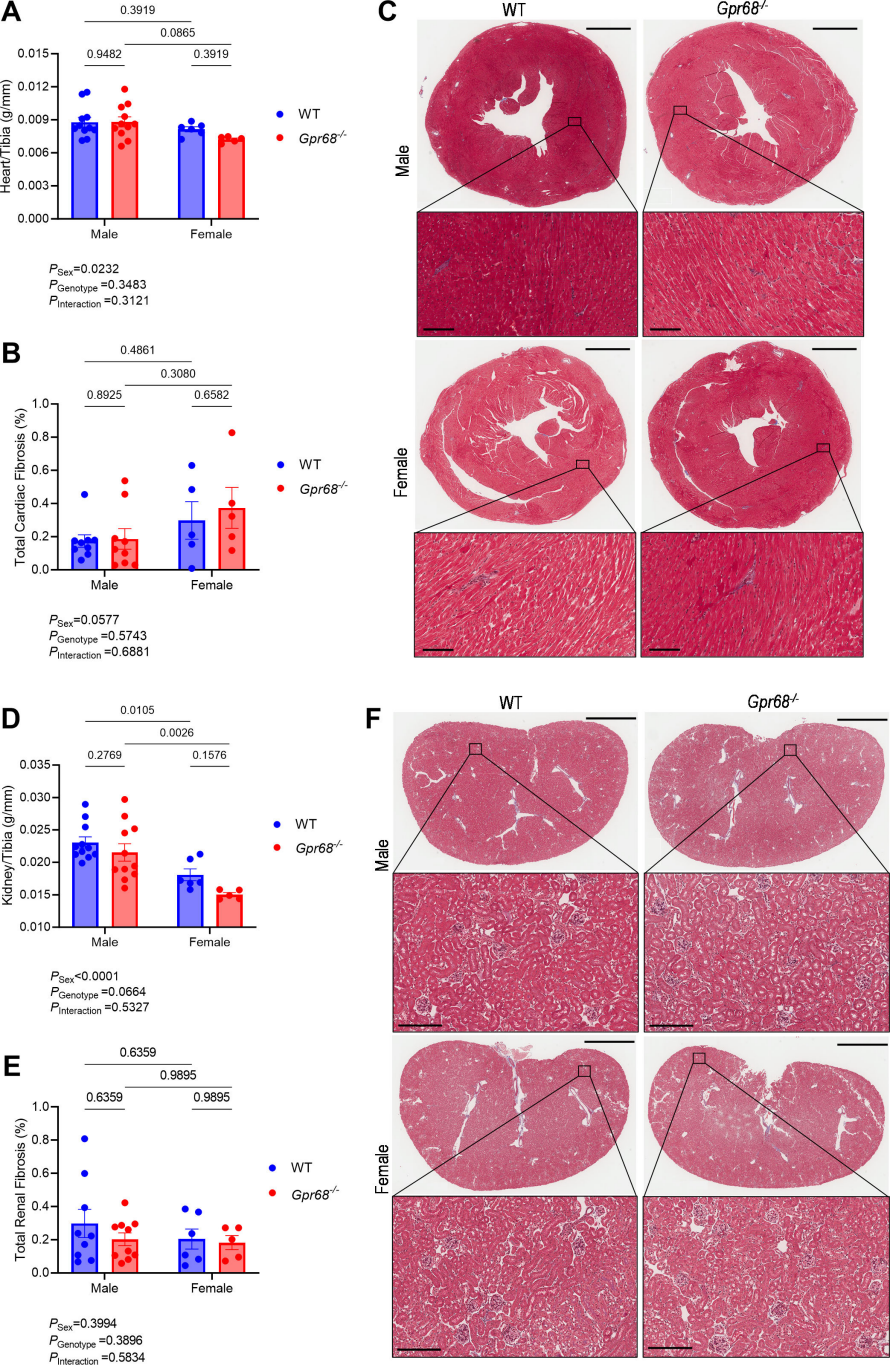
Characterisation of GPR68 deficiency in cardio-renal phenotypes of male and female wildtype (WT) and Gpr68−/− mice. (**A**) Heart weight to tibia length index, (**B**) percentage of total cardiac fibrosis (collagen deposition) and (**C**) Masson’s Trichrome-stained heart sections showing collagen deposition (blue). (**D**) Kidney weight to tibia length index, (**E**) overall percentage of total renal fibrosis (collagen deposition) and (**F**) Masson’s Trichrome-stained kidney sections showing collagen deposition (blue). For representative heart sections, upper panels scale bar=2 mm; for lower panels scale bar=100 μm. For representative kidney sections, upper panels scale bar=2 mm; for lower panels, scale bar=200 μm. Each data point represents an individual sample. Two-way ANOVA with Benjamini and Hochberg’s false discovery rate adjustment for multiple comparisons, showing q-value. Data presented as mean ± SEM. *n*=4–11/group.

We first examined the impact of GPR68 in the cardiovascular system of untreated male and female *Gpr68^−/−^* mice by characterising several key cardiovascular phenotypes. There was no difference in 24-hour baseline BP parameters, including systolic BP, diastolic BP, MAP and heart rate between WT and *Gpr68^−/−^* mice, irrespective of sex (Figures 1A–Cand S4A ). However, *Gpr68^−/−^* mice were more active than WT mice, and females were more active than males over 24 hours (Supplementary Figure S4B). Echocardiography revealed a sex-specific difference in left ventricular end diastolic volume driven by GPR68 deficiency (Figure 1D); however, no differences were observed in ejection fraction output (Figure 1E). Female mice overall had increased left ventricular posterior wall thickness at diastole compared with males, which was driven by GPR68 deficiency (Figure 1F). Similarly, female mice overall had smaller heart weights than males; however, these differences were not driven by GPR68 deficiency (Figure 2A). No difference in total cardiac fibrosis was observed regardless of genotype or sex (Figure 2B and C). These results were consistent in the kidneys (Figure 2D–F). Overall, most sex-specific differences in cardiovascular phenotypes observed were not influenced by the absence of GPR68.

### GPR68 deficiency does not affect the development of hypertension under a control diet

**Figure 3:**
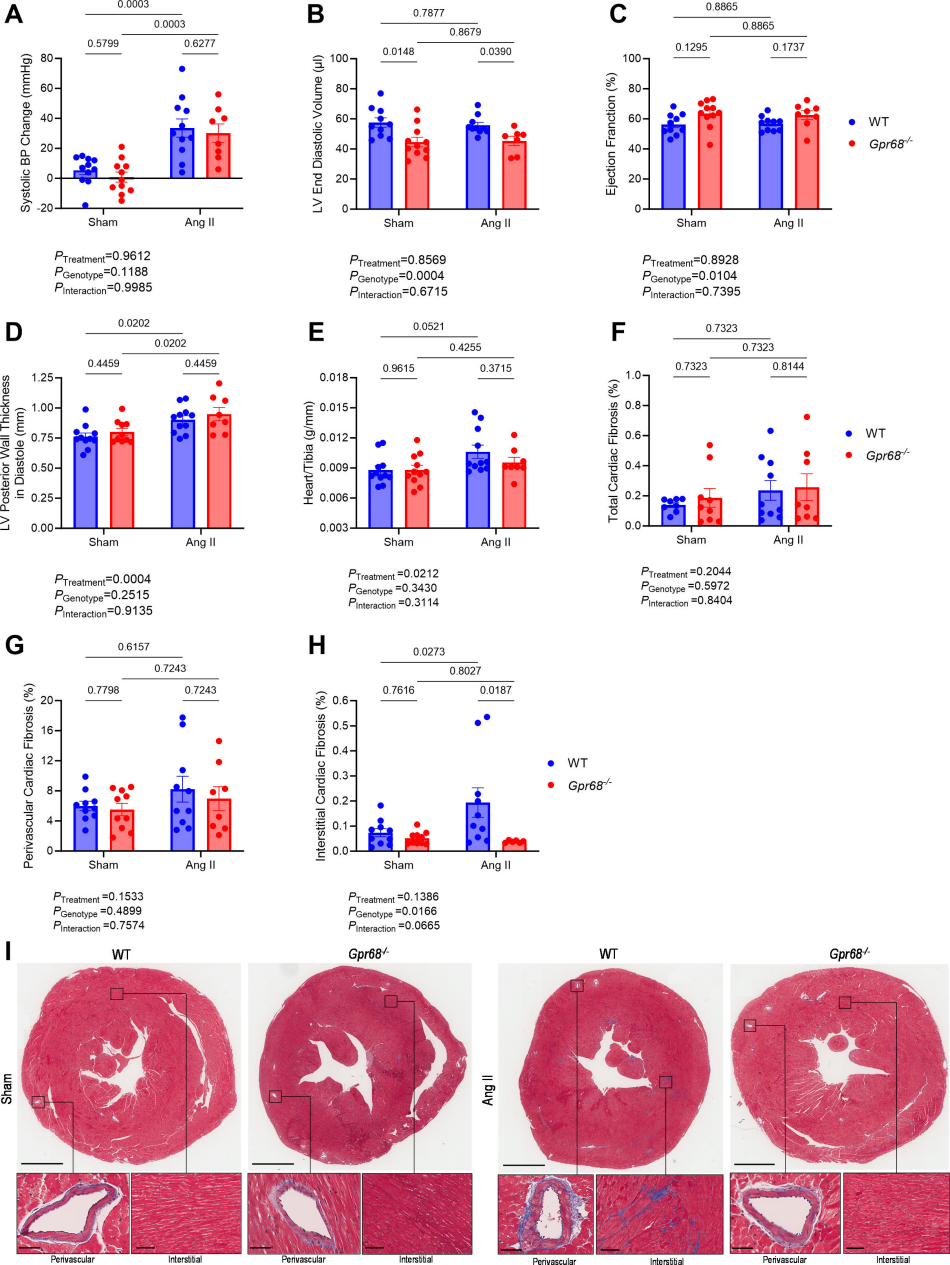
The effect of GPR68 deficiency on blood pressure and cardiac outcomes in Ang II-induced hypertension in male WT and *Gpr68^−/−^* mice. Six-to-eight-week-old mice underwent minipump implantation containing 0.9% sodium chloride (sham) or angiotensin II (Ang II; 0.75 mg/kg body weight/day) released for four weeks. (**A**) Systolic blood pressure (BP) change between baseline and week 4 measurements. (**B**) Left ventricular (LV) end diastolic volume, (**C**) ejection fraction and (**D**) LV posterior wall thickness in diastole obtained from cardiac ultrasounds following the four-week protocol. (**E**) Heart weight to tibia length index. Percentage of (**F**) total, (**G**) perivascular and (**H**) interstitial cardiac fibrosis (collagen deposition; blue). (**I**) Masson’s Trichrome-stained heart sections showing overall, perivascular and interstitial collagen deposition (blue). For representative heart sections, upper panels scale bar=2 mm; for lower panels, perivascular scale bar=50 μm, interstitial scale bar=100 μm. Each data point represents an individual sample. Repetitive measures analyses were used for weekly tail-cuff BP. Two-way ANOVA with Benjamini and Hochberg’s false discovery rate adjustment for multiple comparisons was used for all other data, showing q-values. Data presented as mean ± SEM. *n*=8–11/group.

The role of GPR68 in BP regulation remains largely unknown. Therefore, we first sought to investigate whether the absence of GPR68 affects cardiovascular parameters following a hypertensive insult with Ang II under a control diet (Supplementary Figure S5A). Ang II-treated male WT and *Gpr68^−/−^* mice developed significantly higher systolic BP compared with genotype-matched sham-treated mice (Figures3A and S6and ). GPR68 deficiency also resulted in reduced left ventricular end diastolic volume and subsequent greater ejection fraction regardless of hypertensive insult (Figure 3B and C). Ang II-treated male WT and *Gpr68^−/−^* mice had increased cardiac hypertrophy (shown as left ventricular posterior wall and heart-to-tibia length) compared with sham-treated mice (Figure 3D–E). However, despite no genotype- or treatment-driven differences in total or perivascular cardiac fibrosis (Figure 3F and G), Ang II-treated WT males showed a significant increase in interstitial collagen deposition (Figure 3H and I). Notably, GPR68 deletion did not increase Ang II-induced interstitial cardiac fibrosis in male mice. As GPR68 has also been implicated as a mechanosensor of shear stress in arteries and arterioles [32], we also examined elastin quantity and overall collagen deposition. The percentage of total medial elastin and collagen deposition in the aorta was not influenced by GPR68 deficiency or Ang II treatment (Supplementary Figure S7). *Gpr68^−/−^* male mice had smaller kidneys; Ang II treatment did not affect this (Supplementary Figure S8A). However, there were no changes in renal collagen deposition, irrespective of genotype or treatment (Supplementary Figure S8B and C). We also studied the large intestine of these animals and observed no difference in intestinal weight or length, nor in the number of goblet cells, muscularis propria and collagen thickness (Supplementary Figure S9). Conversely, Ang II-treated female WT and GPR68 KO mice did not develop high BP or associated cardiac changes (Supplementary Figure S11A–C). However, GPR68 deficiency resulted in smaller renal weights (Supplementary Figure S11D) but no associated change in overall renal fibrosis (Supplementary Figure S11E and F).

### GPR68 deficiency reduces dietary fibre effect against Ang II-induced hypertension

**Figure 4:**
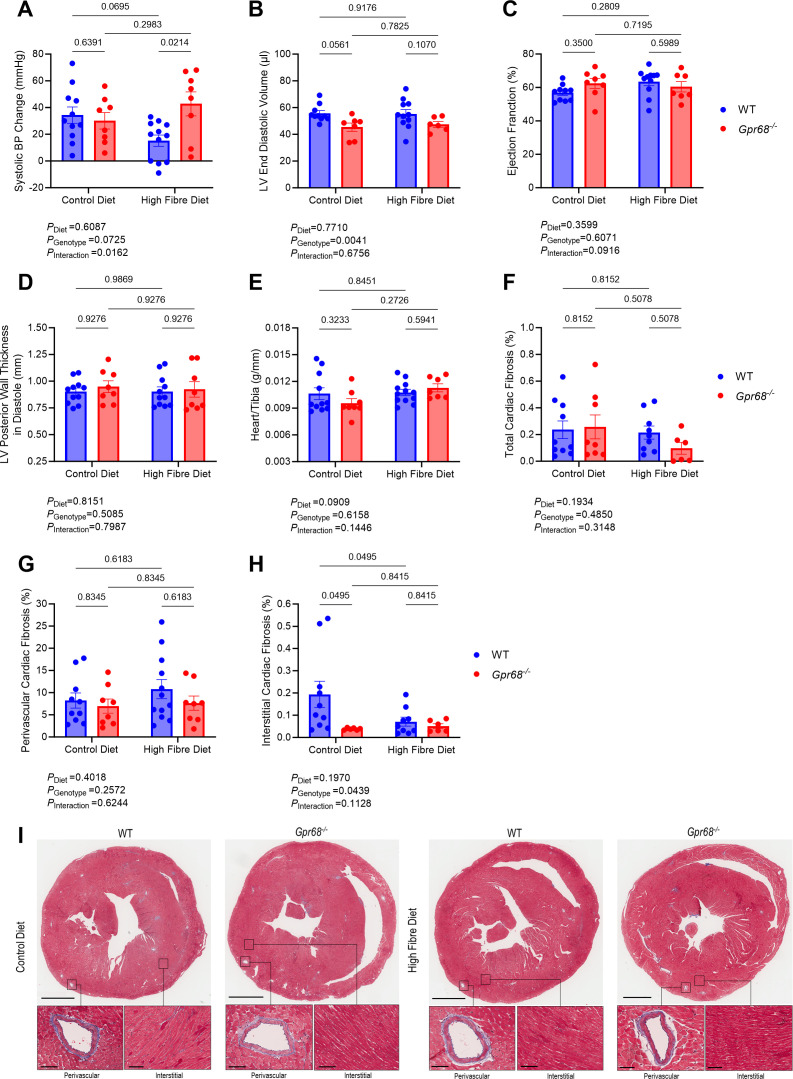
The effect of GPR68 deficiency and dietary fibre on blood pressure and cardiac function in Ang II-induced hypertension in male WT and *Gpr68^−/−^* mice. Six-to-eight-week-old mice underwent minipump implantations containing angiotensin II (Ang II; 0.75 mg/kg body weight/day) and were fed either a control or high-fibre diet for four weeks. (**A**) Systolic blood pressure (BP) change between baseline and week 4 measurements. (**B**) Left ventricular (LV) end diastolic volume, (**C**) ejection fraction and (**D**) LV posterior wall thickness in diastole obtained from cardiac ultrasounds following the four-week protocol. (**E**) Heart weight to tibia length index. Percentage of (**F**) total, (**G**) perivascular and (**H**) interstitial cardiac fibrosis (collagen deposition; blue). (**I**) Masson’s Trichrome-stained heart sections showing overall, perivascular and interstitial collagen deposition (blue). For representative heart sections, upper panels scale bar=2 mm; for lower panels, perivascular scale bar=50 μm, interstitial scale bar=100 μm. Each data point represents an individual sample. Repetitive measures analyses were used for weekly tail-cuff BP. Two-way ANOVA with Benjamini and Hochberg’s false discovery rate adjustment for multiple comparisons was used for all other data, showing q-values. Data presented as mean ± SEM. *n*=6–12/group.

We then sought to investigate whether dietary fibre remained protective under hypertensive conditions without GPR68 (Supplementary Figure S5B). Compared with WT, GPR68-deficient mice fed a high-fibre diet had significantly higher BP (Figure 4A, Supplementary Figure S12). Left ventricular end diastolic volume remained lower in mice lacking GPR68 despite dietary fibre intervention (Figure 4B). Changes in ejection fraction and left ventricular posterior wall thickness were negligible (Supplementary Figure S12C and D). Heart-to-tibia length, total cardiac fibrosis and perivascular fibrosis remained similar independent of diet or genotype (Figure 4E–G); however, interstitial cardiac fibrosis was lower in GPR68 KO mice and was reduced by a high-fibre diet (Figure 4H and I). Dietary fibre did not affect renal weight and total collagen deposition in WT and GPR68 KO mice (Supplementary Figure S13).

### Dietary fibre intake induces histopathological and gut microbial changes in the large intestine independently of GPR68

**Figure 5:**
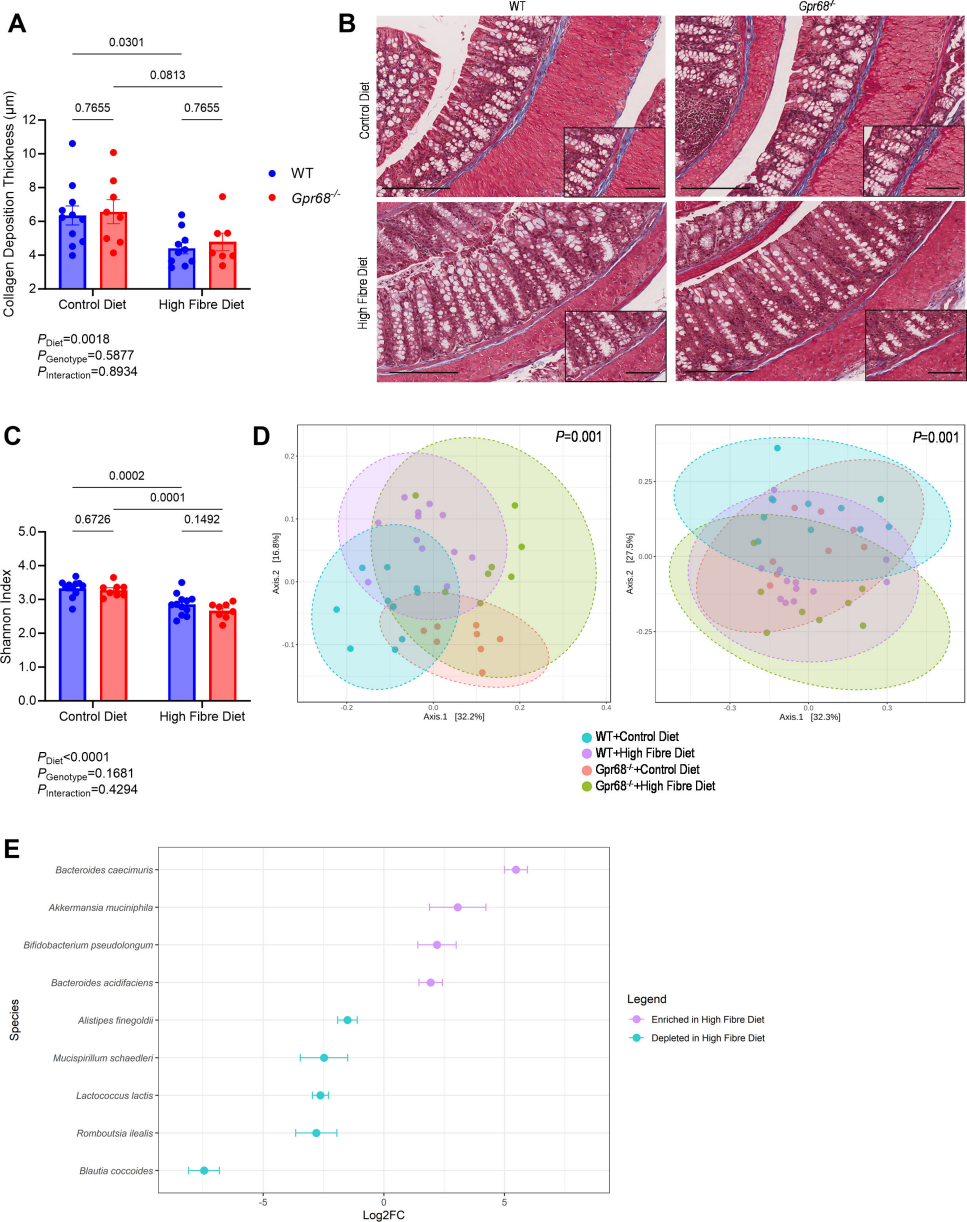
The effect of GPR68 deficiency and dietary fibre on histological and gut microbiota outcomes in the colon and caecal content, respectively, in Ang II-induced hypertensive male WT and Gpr68−/− mice. Six-to-eight-week-old mice underwent minipump implantations containing angiotensin II (Ang II; 0.75 mg/kg body weight/day) and were fed either a control or high-fibre diet for four weeks. (**A**) Quantified colonic collagen deposition thickness (μm). (**B**) Masson’s Trichrome-stained colon sections showing collagen deposition thickness (blue) within the muscularis propria layer. (**C**) α-diversity measured as Shannon diversity index. (**D**) β-diversity shown as unweighted (left plot) and weighted (right plot) UniFrac Principle Coordinate Analysis (PCoA) plots with PERMANOVA *P*-values. (**E**) Abundance levels of nine bacterial species enriched (purple) or depleted (blue) in high-fibre-fed hypertensive mice, showing q-value. For representative colon sections, zoomed out image scale bar=200 μm, zoomed in image scale bar=100 μm. Each data point represents an individual sample. Pairwise PERMANOVA analysis was performed on unweighted and weighted UniFrac metrics. Two-way ANOVA with Benjamini and Hochberg’s false discovery rate adjustment for multiple comparisons was used for all other data, showing q-value. Data presented as mean ± SEM. Differentially abundant bacterial taxa data presented as log-transformed count; box plot data presented as median and interquartile range. *n*=8–12/group.

The gut is responsible for maintaining the balance between immune activation and the gut microbiota [16]. Therefore, we examined morphological markers of large intestinal tissue in response to a hypertensive stimulus and varying levels of fibre intake and its effects on the gut microbiota. We found that hypertensive mice on a high-fibre diet had heavier large intestines and a significantly longer colon than mice on a control diet, independent of GPR68 (Supplementary Figure S14A and B). Dietary fibre also reduced collagen deposition within the muscularis propria layer of hypertensive mice, regardless of GPR68 (Figure 5A and B). However, there were no diet- or genotype-driven differences in the number of goblet cells or overall thickness of the muscularis propria layer (Supplementary Figure S14C–E). We then examined the gut microbiota composition by sequencing the bacterial 16S rRNA gene from caecal content. Samples were rarefied to a minimum library size of 10,875 reads, plateauing species richness (Supplementary Figure S15). Dietary fibre reduced the Shannon diversity index, a metric of α-diversity, an effect observed independently of GPR68 (Figure 5C). Hypertensive mice fed a control or high-fibre diet had distinctly different gut microbial signatures that were not influenced by GPR68 (Figure 5D and S16). These data were validated using multiple linear regression analysis with covariate adjustment, which allowed us to control for genotype. Indeed, diet remained the primary causative factor in differentially abundant species. High-fibre-fed hypertensive mice had a lower abundance of *Blautia coccoides*, *Lactococcus lactis* and *Alistipes finegoldii*, but greater abundances of *Bacteroides acidifaciens* and *Akkermansia muciniphila* (Figure 5E). Additionally, bacterial species unique to the rodent microbiome were identified, including *Romboutsia ilealis*, which was depleted in high-fibre-fed hypertensive mice, as well as *Bacteroides caecimuris* and *Bifidobacterium pseudolongum*, which were enriched in high-fibre-fed hypertensive mice (Figure 5E).

### GPR68 may mediate the effects of dietary fibre through the vasculature, rather than the immune system, in Ang II mice

**Figure 6:**
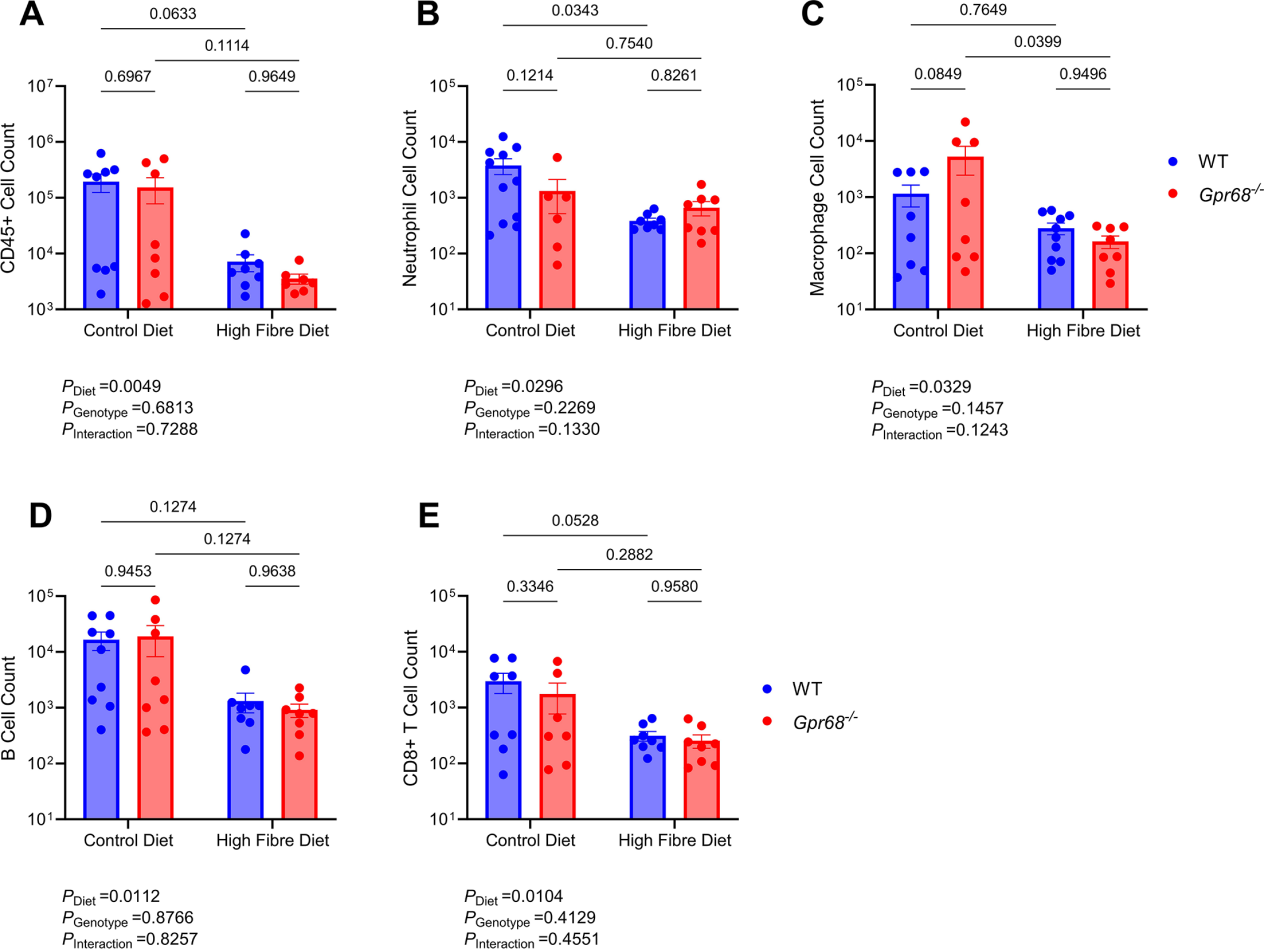
The effect of GPR68 deficiency and dietary fibre on immune cell population counts in the aorta of Ang II-induced hypertensive male WT and *Gpr68^−/−^* mice. Six-to-eight-week-old mice underwent minipump implantations containing angiotensin II (Ang II; 0.75 mg/kg body weight/day) and were fed either a control or high-fibre diet for four weeks. Flow cytometric analyses were performed on aortic single-cell suspensions following the four-week protocol. (**A**) Overall immune (CD45^+^), (**B**) neutrophil, (**C**) macrophage, (**D**) B- and (**E**) CD8^+^T cell counts. Each data point represents an individual sample. Two-way ANOVA with Benjamini and Hochberg’s false discovery rate adjustment for multiple comparisons, showing q-value. Data presented as mean ± SEM. *n*=8–11/group.

**Figure 7:**
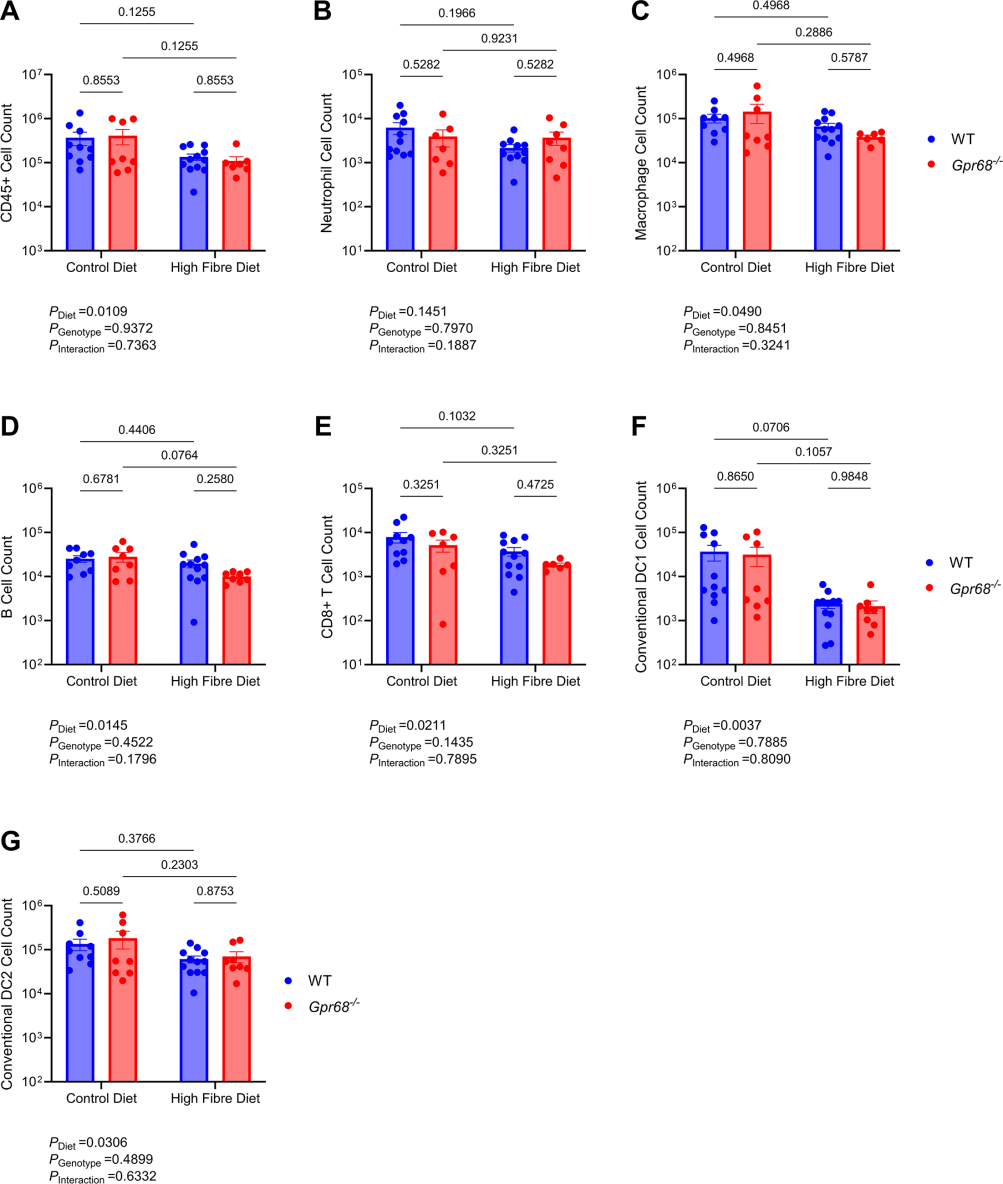
The effect of GPR68 deficiency and dietary fibre on immune cell population counts in the kidney of Ang II-induced hypertensive male WT and *Gpr68^−/−^* mice. Six-to-eight-week-old mice underwent minipump implantations containing angiotensin II (Ang II; 0.75 mg/kg body weight/day) and were fed either a control or high-fibre diet for four weeks. Flow cytometric analyses were performed on kidney single-cell suspensions following the four-week protocol. (**A**) Overall immune (CD45^+^), (**B**) neutrophil, (**C**) macrophage, (**D**) B-, (**E**) CD8^+^T, (**F**) type 1 and (**G**) type 2 conventional dendritic cell (cDC1; cDC2) counts. Each data point represents an individual sample. Two-way ANOVA with Benjamini and Hochberg’s false discovery rate adjustment for multiple comparisons, showing q-value. Data presented as mean ± SEM. *n*=8–12/group.

**Figure 8:**
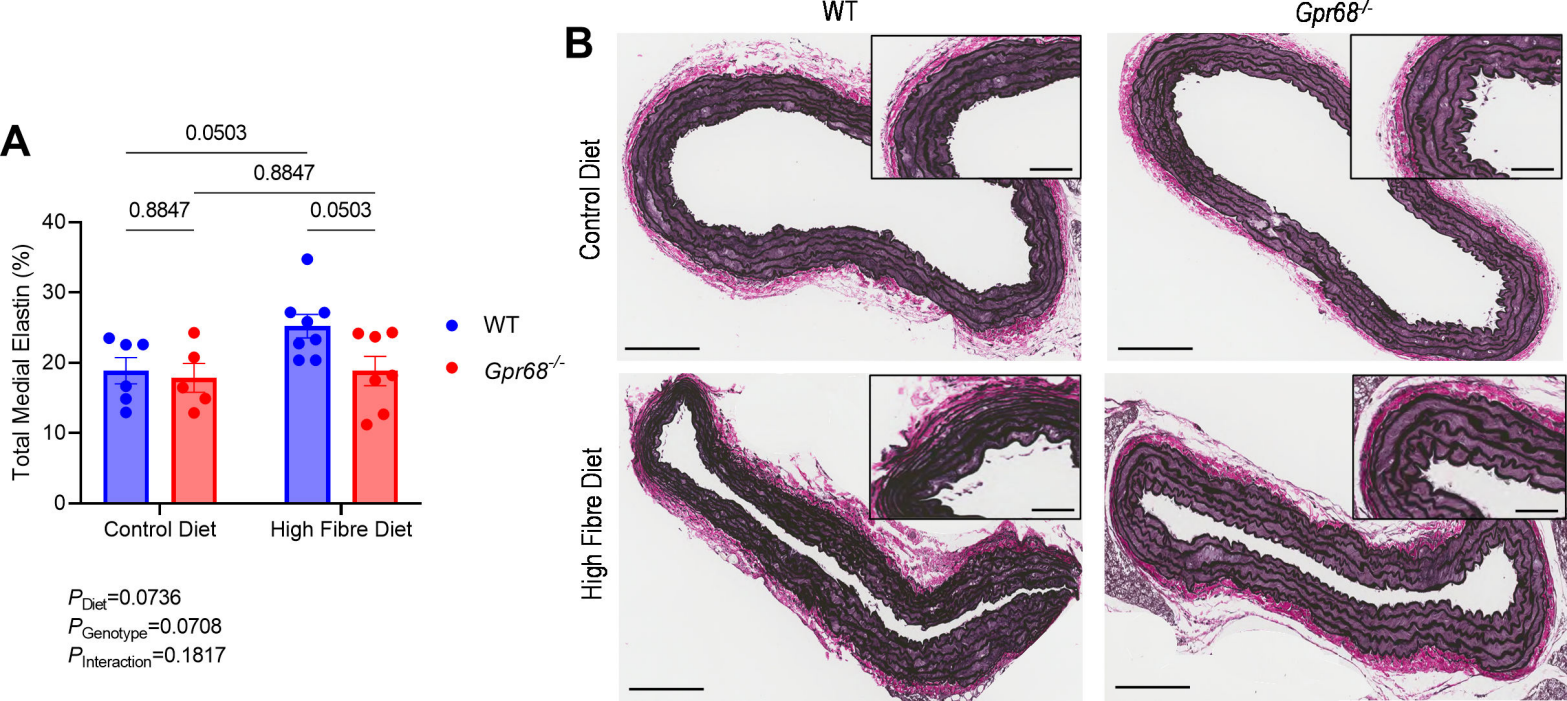
The effect of GPR68 deficiency and dietary fibre on elastin integrity in the aorta of Ang II-induced hypertensive male WT and *Gpr68^−/−^* mice. Six-to-eight-week-old mice underwent minipump implantations containing angiotensin II (Ang II; 0.75 mg/kg body weight/day) and were fed either a control or high-fibre diet for four weeks. (**A**) Total medial elastin content quantified in the aorta. (**B**) Verhoeff–Van Gieson-stained aortic sections showing elastin deposition following the four-week protocol. For representative aorta sections, zoomed out image scale bar=200 μm, zoomed in image scale bar=50 μm. Each data point represents an individual sample. Two-way ANOVA with Benjamini and Hochberg’s false discovery rate adjustment for multiple comparisons, showing q-value. Data presented as mean ± SEM. *n*=8–11/group.

Accumulating evidence supports the role of the immune system in hypertension [12,13]. GPR68, predominantly expressed in immune cells [32], is crucial in mediating inflammatory processes [31,37]. Therefore, as fibre did not restore Ang II-induced high BP in mice lacking GPR68, we first hypothesised that this strain may have exaggerated pro-inflammatory immune cell activation. Altogether, we examined up to 13 immune cell populations within the myeloid and lymphoid lineages; these were not different between WT and GPR68 KO mice (Supplementary Tables S6–S9). However, there was a diet-driven decrease in immune cell counts in the thoracic aorta and kidney, independent of GPR68 (Figures 6 and 7). High-fibre diet reduced the total number of immune cells, neutrophils, macrophages, B cells and CD8^+^T cells in the thoracic aorta, independent of the genotype (Figure 6, Supplementary Table S10). High-fibre-fed mice had significantly reduced renal immune cells, including neutrophils, macrophages, B cells, CD8^+^T cells and type 1 and 2 conventional dendritic cells (Figure 7). Additionally, there was a non-significant reduction in the other immune cell populations examined (Supplementary Table S11). Dietary fibre did not influence immune cell counts in the spleen and peripheral blood (Supplementary Tables S12–S13). Conversely, vascular remodelling, such as in the aorta, is a well-known hallmark of hypertension and is closely linked to the loss of elastin fibre integrity within the aortic vessel wall [55,56]. Whilst aortic collagen deposition remained unchanged (Supplementary Figure S17), hypertensive WT mice fed a high-fibre diet had improvements in overall aortic medial elastin content, whilst this improvement was not observed in hypertensive high-fibre-fed Gpr68^−^/^−^ mice (Figure 8).

## Discussion

BP control remains a global challenge, contributing significantly to CVD risk and mortality [57]. Additionally, suboptimal dietary intake is an essential but preventable risk factor for hypertension and CVDs [57,58]. Recently, the bioactive metabolites released from fibre fermentation have shown promising potential as therapeutic agents for treating experimental and clinical models of hypertension [8,10,11]. SCFAs are recognised for their immunomodulatory effects, which are mediated through the activation of metabolite-sensing GPCRs [59]. A large body of work has pinpointed three specific SCFA-sensing GPCRs that are constitutively expressed on immune cells and implicated in cardiovascular protection (i.e. GPR41, GPR43 and GPR109A) [19–21,60]. In this study, we provide evidence suggesting that pH-sensing GPR68 is involved in the underlying mechanisms conferring the BP-lowering nature of dietary fibre and its associated metabolites independently of these receptors. Due to the gut microbiota’s essential role in priming the immune system and GPR68’s high expression in immune cells, we investigated whether this mechanism could be immune-driven. We provide robust evidence suggesting that dietary fibre reduces pro-inflammatory immune cell infiltration into the kidney and the thoracic aorta; however, these were independent of GPR68.

Our study demonstrates that GPR68 partially mediates the cardiovascular protection (BP and cardiac interstitial collagen deposition) conferred by dietary fibre and that this is independent of the immune system. Thus, other mechanisms may be at play. A seminal study found that, in addition to sensing extracellular protons, GPR68 has also played an essential role as a mechanosensor of shear stress in human and mouse vascular endothelial cells [32]. Hypertension is also known to be associated with the disruption and eventual loss of elastin fibres within the aortic vessel wall [55]. Interestingly, we reported that high-fibre-fed WT male mice exhibited greater medial elastin content than control diet-fed WT male mice. However, in the absence of GPR68, high dietary fibre failed to improve the percentage of medial elastin content in hypertensive mice. This suggests that GPR68 may mediate the protective effects of dietary fibre by preserving aortic elastin integrity, and possibly overall vascular integrity, under hypertensive conditions. A recent study supports this hypothesis, whereby authors reported exaggerated medial wall remodelling in GPR68-knockout vessels [34]. Vascular smooth muscle cells constitute the main cell type lining the medial layer of the arterial wall [61]. These cells are the principal mediators of elastin synthesis, secretion and deposition [62] and notably express GPR68 [32], suggesting a potential role for this receptor in maintaining regulatory mechanisms within the extracellular matrix of the aorta.

GPR68 has previously been implicated in immune cell regulation under acidic microenvironments [36]. For example, GPR68-deficient mice have significantly higher infiltrating lymphocytes in tumour tissue [63]. The role of the immune system has been increasingly recognised as a key contributor to the development and progression of hypertension [12,13,64], thus making hypertension an unconventional inflammatory disease. As GPR68 is particularly enriched in immune cells [29,30], we explored whether the immune system mediates the mechanistic link between GPR68 and dietary fibre in hypertension. However, our data revealed that the effects of dietary fibre on the immune system were mostly independent of GPR68. For example, macrophage cell counts were significantly lower in the kidney and aorta of high-fibre-fed mice. Macrophages are antigen-presenting cells that promote the recruitment of other immune cells, predominantly T and B cells, to sites of inflammation [65]. This is consistent with our data, which shows a reduction in B and CD8^+^T cells, both well-known immune cell subsets that play an active role in the pathogenesis of hypertension [66,67]. We also identified lower renal infiltration of specific subsets of dendritic cells in high-fibre-fed mice. Dendritic cells are the most potent antigen-presenting cells that activate T cells and arguably play a fundamental role in immune-mediated BP elevation [40,68,69]. Specifically, we identified type 1 conventional dendritic cells, which excel in cross-presentation of antigens, and type 2 conventional dendritic cells, typically involved in CD4^+^T cell induction [70].

Moreover, our findings revealed that dietary fibre, rather than GPR68, was the primary factor influencing differences in large intestinal physiology and the gut microbiota of hypertensive mice. This is consistent with previous studies showing that diet had a larger influence on the microbiota than genotype or Ang II [71], and clinical studies showing that dietary fibre modulates the microbiota [11,72]. Recent evidence also supports the role of the gut microbiota in BP regulation, with numerous studies implicating alterations in the gut microbiota, termed gut dysbiosis, in the development and progression of hypertension [73]. For example, human gut microbiome studies have reported an association between higher abundances of gram-negative bacteria and high BP [74,75], including *Alistipes finegoldii*, which we found to be more abundant in control-fed hypertensive mice. However, we also observed greater abundances of gram-positive bacterial species including *Blautia coccoides,* which has been previously associated with glucose intolerance and type 2 diabetes mellitus [76]. This bacterial strain has also been linked to the activation of tumour necrosis factor-α [77], a pro-inflammatory cytokine mainly secreted by macrophages [13,78]. Notably, we observed increased levels of macrophages in the aorta and kidney of control-fed hypertensive mice. Moreover, an increasing amount of data has highlighted gut dysbiosis as an intrinsic link with the development of fibrosis in several organs [79] and in patients with heart failure with preserved ejection fraction [80], which may explain the excess collagen deposition observed in control-fed hypertensive colonic tissue. Moreover, *Bacteroides* species are the most predominant anaerobes in the gut and have associated cardiovascular benefits [81,82]. Interestingly, *Bacteroides* spp. are among the primary SCFA producers [83], with optimal fermentation occurring at pH levels between 6.0 and 7.0—the typical caecum and colon luminal pH [84,85], which could activate GPR68 and other pH-sensing GPCRs such as GPR65 [28].

## Limitations

We acknowledge our study had limitations. Ablation of GPR68 resulted in smaller kidney weights in both male and female mice, a characteristic that may be attributed to the fact that GPR68 is expressed on renal tubule epithelial cells [33]. While direct correlations between GPR68 expression and kidney size via tubule epithelial cells have not been widely established, there is growing evidence suggesting that GPR68 may play a role in the regulation of epithelial cell behaviour and homeostasis [86,87], which could, in turn, influence kidney morphology and function. Therefore, future studies should consider undertaking comprehensive metabolic and renal function assessments of these animals, such as metabolic caging and body composition profiling, to provide a more integrated understanding of the physiological effects of GPR68.

WT female mice did not develop higher BP when challenged with Ang II, and GPR68 did not sensitise female mice to Ang II-induced hypertension. This is not entirely unexpected, as clinical and experimental studies in females have shown that sex hormones, particularly estrogen and progesterone, are cardioprotective before and after menopause [88–90]. The protective effects of sex hormones may also override the role of GPR68 in BP regulation. A future direction could involve using aged female mice or exposure to a longer Ang II infusion to elicit a response. This approach would help determine whether there is a sex-specific phenotype for high-fibre diets or the role of GPR68 in BP control. Understanding these nuances could provide valuable insights into sex differences in hypertension and the cardioprotective mechanisms of dietary fibre interventions.

We acknowledge that the use of a global knockout model for GPR68 is a limitation of the current study. Tissue-specific knockouts, such as intestinal epithelial knockouts, may be relevant to further detailing how GPR68 may regulate BP during fibre intake. There is robust evidence demonstrating that overexpression of GPR68 in the human intestinal epithelial cell line, Caco-2, led to pH-dependent activation of numerous signalling pathways [91]. For example, inositol phosphate accumulation and calcium mobilisation are key downstream signalling events involved in the maintenance of epithelial integrity [91–93], while serum response factor-dependent transcription regulates genes associated with gut barrier function [91,94]. Therefore, whilst other pH-sensing receptors may partially compensate for the loss of GPR68, collectively, these signalling pathways indicate that GPR68 contributes to the ability of intestinal epithelial cells to sense and respond to changes in extracellular pH, thereby potentially regulating gut barrier integrity and mucosal adaptation to dietary cues.

Nonetheless, other pH-sensing and regulatory mechanisms—including those involving transient receptor potential vanilloid 1 (TRPV1) and acid-sensing ion channels (ASICs)—cannot be overlooked. TRPV1 is abundantly expressed in immune cells [95,96], including resident colonic immune cells, particularly CD4^+^T cells [97,98], and has been implicated in the underlying pathogenesis of T cell-mediated murine colitis [98]. Similar to TRPV1, ASICs were first identified in sensory neurons and were found to mediate pain perception [99]. Unlike TRPV1, however, their role in inflammatory conditions such as GI inflammation has been attributed to neuronal expression and activation [100], despite evidence that certain subsets of immune cells also express these channels [101,102]. Therefore, these alternative pH-sensing pathways may also be contributing to the immunomodulatory and overall protective effects of dietary fibre, highlighting a broader network of pH-responsive mechanisms under hypertensive conditions. Our results also suggest a potential protective role for GPR68 in the vasculature, specifically in supporting elastin maintenance within the aortic vessel wall. While promising, these observations require further validation; for example, vascular mechanics measurements should be measured to explore vascular functional changes linked to Gpr68 activation. Nonetheless, the aorta is not a resistance artery and, therefore, does not directly regulate BP [103]. As such, further studies are needed to characterise the role of GPR68 in arterial function, such as pulse wave velocity studies.

## Conclusions

Here, we leveraged the understanding of how SCFAs influence colonic pH to investigate whether pH-sensor GPR68 underpins the BP-lowering effects of dietary fibre. Whilst our findings suggest that GPR68 contributes to these effects, they are most likely mediated independently of the immune system, warranting further investigation into its underlying mechanism of action.

**Clinical Perspectives**

- Dietary fibre lowers blood pressure (BP) via gut microbial metabolites, which produce acidic metabolites that lower large intestinal pH. This could activate signalling via pH-sensing receptors, such as GPR68. Targeting the pH-sensing receptor GPR68 may offer a new approach for leveraging the BP-lowering effects of dietary fibre in hypertension.
- This study demonstrates that the pH-sensing receptor GPR68 partially mediates how dietary fibre lowers BP in a pre-clinical model of hypertension. Dietary fibre reduced pro-inflammatory immune cell infiltration into the kidney and the thoracic aorta, and influenced differences in large intestinal physiology and the gut microbiota of hypertensive mice, all independently of GPR68.
- Strategies to increase dietary fibre intake may have an anti-inflammatory effect, most likely independently of GPR68. Nonetheless, personalised nutritional strategies tailored to optimise SCFA production could be explored as an adjunctive or preventative measure for patients at risk of, or suffering from, hypertension.

## Supplementary material

Online supplementary material 1

## Data Availability

Sequencing data and metadata file are publicly available at https://doi.org/10.5281/zenodo.15522771.

## Abbreviations

ASIC: acid-sensing ion channels
ASV: amplicon sequencing variant
Ang II: angiotensin II
BP: blood pressure
CVD: cardiovascular disease
FDR: false discovery rate
GI: gastrointestinal
GPCR: G-protein coupled receptor
MAP: mean arterial pressure
NK: natural killer
PCR: polymerase chain reaction
PCoA: Principal Coordinate Analysis
PERMANOVA: permutational multivariate analysis of variance
SCFAs: short-chain fatty acids
TRPV1: transient receptor potential vanilloid 1
WT: wildtype
crRNA: CRISPR RNA
rRNA: ribosomal RNA
tracrRNA: trans-activating crRNA
