# Supplementary material for: pH-sensor GPR68 plays a role in how dietary fibre lowers blood pressure in a preclinical model of hypertension
Source: Clin Sci (Lond). 2025 Oct 28;139(21):1239–59. doi: 10.1042/CS20243009 (PMC12687442; doi:10.1042/CS20243009)
Supplement: Online supplementary material 1 [file cs-139-21-CS20243009-s001.docx]

**Online supplementary files to**

**pH-sensor GPR68 plays a role in how dietary fibre lowers blood pressure in a preclinical model of hypertension**

Evany Dinakis,^1^ Chudan Xu,^1^ Rikeish R Muralitharan,^1,2^ Hamdi Jama,^1^ Liang Xie,^1,3,4^ Kwan Charmaine Leung,^1^ Katrina M. Mirabito Colafella,^5^ Zoe McArdle,^5^ Ekaterina Salimova,^6^ Leticia Camargo Tavares,^1^ Matthew Snelson,^1,2^ Chad Johnson,^7^ Tracey Gaspari,^8^ Charles R. Mackay,^3,9^ Joanne A. O’Donnell,^1^ Francine Z. Marques^1,2,10*^

^1^ Hypertension Research Laboratory, Department of Pharmacology, Biomedical Discovery Institute, Faculty of Medicine, Nursing and Health Sciences, Monash University, Clayton, Australia; ^2^Victorian Heart Institute, Monash University, Melbourne, Australia;^3^Department of Microbiology, Monash Biomedicine Discovery Institute, Monash University, Melbourne, Australia; ^4^Precision Medicine Translational Research Programme, Department of Obstetrics & Gynaecology, Yong Loo Lin School of Medicine, National University of Singapore, Singapore; ^5^Cardiovascular Disease Program, Department of Physiology, Monash Biomedicine Discovery Institute, Monash University, Melbourne, Australia; ^6^Monash Bioimaging Facility, Monash University, Melbourne, Australia; ^7^Bioimaging Platform, La Trobe University, Melbourne, Australia; ^8^IRAP Pharmacology Group, Department of Pharmacology, Monash Biomedicine Discovery Institute, Monash University, Melbourne, Australia; ^9^School of Pharmaceutical Sciences, Shandong Analysis and Test Center, Qilu University of Technology (Shandong Academy of Sciences), Jinan, 250014, China. ^10^Baker Heart and Diabetes Institute, Melbourne, Australia.

***Corresponding author**: Prof Francine Marques, Hypertension Research Laboratory, Victorian Heart Institute, Level 2, Victorian Heart Hospital, 631 Blackburn Road Clayton, VIC 3168 Monash University, Melbourne, Australia, Phone: +61-03-7511 1864. E-mail: [francine.marques@monash.edu](mailto:francine.marques@monash.edu)

**Supplementary tables**

**Table S1.** Summary table of experimental animal usage.

| **Sex** | **Genotype** | **Age (Weeks)** | **No. of animals** | **Procedure** | **Dietary Intervention** |
| --- | --- | --- | --- | --- | --- |
| **Male** | **WT** | 8-10 | 6 | Baseline telemetry | None |
|  |  | 6-8 | 11 | Minipump surgery; sham | Control |
|  |  | 6-8 | 11 | Minipump surgery; Ang II | Control |
|  |  | 6-8 | 12 | Minipump surgery; Ang II | High-fibre |
| **Total** |  |  | 40 |  |  |
| **Male** | ***Gpr68^-/-^*** | 8-10 | 4 | Baseline telemetry | N/A |
|  |  | 6-8 | 11 | Minipump surgery; sham | Control |
|  |  | 6-8 | 8 | Minipump surgery; Ang II | Control |
|  |  | 6-8 | 8 | Minipump surgery; Ang II | High-fibre |
| **Total** |  |  | 31 |  |  |
| **Female** | **WT** | 8-10 | 6 | Baseline telemetry | N/A |
|  |  | 6-8 | 6 | Minipump surgery; sham | Control |
|  |  | 6-8 | 4 | Minipump surgery; Ang II | Control |
| **Total** |  |  | 16 |  |  |
| **Female** | ***Gpr68^-/-^*** | 8-10 | 4 | Baseline telemetry | N/A |
|  |  | 6-8 | 5 | Minipump surgery; sham | Control |
|  |  | 6-8 | 4 | Minipump surgery; Ang II | Control |
| **Total** |  |  | 13 |  |  |
| **Overall total** |  |  | 100 |  |  |

**Table S2.** Nutrient breakdown of diets used in study (supplied by Speciality Feeds).

|  | **Control** | **High Fibre** |
| --- | --- | --- |
| **Crude fibre** | 4.7% | 9.7 % ^*^ |
| **Acid detergent fibre** | 4.7% | 9.7 % ^*^ |
| **Protein** | 19.4% | 19.4 % |
| **Sodium** | 0.15% | 0.12% |
| **Total Fat** | 7.0% | 7.0% |
| **% Total calculated digestible energy from lipids** | 16.0% | 16.4% |
| **% Total calculated digestible energy from protein** | 21.0% | 21.9% |
| **% Total calculated digestible energy from carbohydrates** | 56.8% | 61.7% ^*^ |
| **Digestible energy** | 16.1 MJ/Kg | 15.6 MJ/Kg |

^*^All carbohydrates replaced with Gel Crisp Starch (636 g/Kg); Gel Crisp is a modified high amylose starch sourced from maize starch

**Table S3.** Myeloid and B-cell surface antibody panel for peripheral blood, spleen and kidney single-cell suspension flow cytometric staining.

| **Marker** | **Fluorophore** | **Clone** | **Manufacturer** | **Dilution** |
| --- | --- | --- | --- | --- |
| F4/80 | APC-Cy7 | BM8 | Biolegend | 1:100 |
| CD11c | BV605 | N418 | Biolegend | 1:100 |
| CD86 | BV780 | GL-1 | Biolegend | 1:100 |
| MHC Class II | PerCP | M5/114.15.2 | Biolegend | 1:100 |
| CD11b | BV421 | M1/70 | Biolegend | 1:200 |
| Ly6G | PeCy7 | 1A8 | Biolegend | 1:500 |
| CD45 | AF700 | 30-F11 | Biolegend | 1:500 |
| Ly6C | FitC | HK1.4 | Biolegend | 1:1000 |
| B220 | PE | RA3-6B2 | Biolegend | 1:1000 |

**Table S4.** T-cell surface and intracellular antibody panel for peripheral blood, spleen and kidney single-cell suspension flow cytometric staining.

| **Marker** | **Fluorophore** | **Clone** | **Manufacturer** | **Dilution** |
| --- | --- | --- | --- | --- |
| Foxp3 | PE | FJK-16s | eBioscience | 1:100 |
| TCR-γδ | BV421 | GL3 | Biolegend | 1:100 |
| CCR6 | PECy7 | 29-2L17 | Biolegend | 1:200 |
| NK1.1 | PeCy5 | PK136 | Biolegend | 1:200 |
| CD4 | BV605 | RM4-5 | Biolegend | 1:500 |
| CD8a | PercP Cy5.5 | 53-6.7 | Biolegend | 1:500 |
| CD45 | AF700 | 30-F11 | Biolegend | 1:500 |
| TCR-β | FITC | H57-597 | Biolegend | 1:800 |
| CD44 | BV780 | IM7 | Biolegend | 1:1000 |

**Table S5.** Surface antibody panel for thoracic aortic single-cell suspension flow cytometric staining.

| **Marker** | **Fluorophore** | **Clone** | **Manufacturer** | **Dilution** |
| --- | --- | --- | --- | --- |
| F4/80 | APC-Cy7 | BM8 | Biolegend | 1:100 |
| NK1.1 | PeCy5 | PK136 | Biolegend | 1:200 |
| CD11b | BV421 | M1/70 | Biolegend | 1:200 |
| CD4 | BV605 | RM4-5 | Biolegend | 1:500 |
| CD8a | PercP Cy5.5 | 53-6.7 | Biolegend | 1:500 |
| CD45 | AF700 | 30-F11 | Biolegend | 1:500 |
| Ly6G | PeCy7 | 1A8 | Biolegend | 1:500 |
| Ly6C | FitC | HK1.4 | Biolegend | 1:1000 |
| B220 | PE | RA3-6B2 | Biolegend | 1:1000 |

**Table S6.** Flow cytometric data of the aorta from sham- and Ang II-challenged (0.75mg/kg body weight/day) wild-type (WT) and GPR68-deficient (*Gpr68*^-/-^) male mice.

| **Cell type** | **WT Sham** | **WT Ang II** | ***Gpr68*^-/-^ Sham** | ***Gpr68*^-/-^ Ang II** | ***P*-value** |
| --- | --- | --- | --- | --- | --- |
| **Immune Cells** | 8.702±3.331 | 51.56±22.50 | 19.97±9.517 | 15.26±7.492 | 0.434 |
| **Macrophages** | 0.281±0.131 | 0.469±0.187 | 1.005±0.667 | 0.526±0.279 | 0.891 |
| **Neutrophils** | 0.482±0.190 | 0.380±0.121 | 0.853±0.464 | 0.958±0.582 | 0.985 |
| **B Cells** | 0.980±0.315 | 10.61±7.526 | 2.040±0.873 | 1.892±1.067 | 0.597 |
| **CD4+ T Cells** | 0.211±0.079 | 5.231±3.266 | 0.612±0.282 | 0.633±0.359 | 0.579 |
| **CD8+ T Cells** | 0.185±0.058 | 4.059±2.997 | 0.360±0.164 | 0.414±0.253 | 0.483 |
| **Natural Killer Cells** | 0.008±0.005 | 0.632±0.391 | 0.012±0.007 | 0.006±0.003 | 0.439 |

Normal distribution of data was assessed using Shapiro-Wilk's normality test. Kruskal-Wallis test was performed using GraphPad Prism 9.3.1 for non-normally distributed data. *P*<0.05 was considered statistically significant. Data presented as mean ± SEM (x10^4^) cell counts unless otherwise stated. n=4-11/group.

**Table S7.** Flow cytometric data, including immune cell counts and activation, of the kidney from sham- and Ang II-challenged (0.75mg/kg body weight/day) wild-type (WT) and GPR68-deficient (Gpr68^-/-^) male mice.

| **Cell type** | **WT Sham** | **WT Ang II** | ***Gpr68*^-/-^ Sham** | ***Gpr68*^-/-^ Ang II** | ***P*-value** |
| --- | --- | --- | --- | --- | --- |
| **Immune Cells** | 79.77±37.55 | 55.41±21.78 | 64.17±18.21 | 40.94±15.60 | 0.4329 |
| **Macrophages** | 38.76±20.88 | 26.73±11.99 | 27.06±8.210 | 14.45±6.733 | 0.3469 |
| **Inflammatory Monocytes** | 0.854±0.428 | 0.592±0.252 | 0.741±0.230 | 0.389±0.125 | 0.7247 |
| **Neutrophils** | 0.619±0.222 | 0.623±0.187 | 1.122±0.308 | 0.622±0.269 | 0.3677 |
| **Dendritic Cells** | 37.04±20.53 | 25.94±11.13 | 25.86±7.107 | 13.02±5.403 | 0.3724 |
|  |  |  |  |  |  |
| **cDC1** | 5.830±2.460 | 3.673±1.432 | 5.194±2.055 | 3.147±1.465 | 0.9103 |
| **cDC2** | 45.36±22.87 | 31.13±13.26 | 35.04±10.52 | 18.24±7.918 | 0.3665 |
| **B Cells** | 7.091±3.574 | 6.478±2.749 | 5.072±1.426 | 2.808±0.693 | 0.6878 |
|  |  |  |  |  |  |
|  |  |  |  |  |  |
| **γδ T Cells** | 32.77±13.90 | 28.81±12.02 | 37.68±13.32 | 15.02±6.507 | 0.4244 |
| **CD4+ T Cells** | 2.823±1.337 | 2.400±1.052 | 4.074±1.464 | 1.808±0.741 | 0.5335 |
|  |  |  |  |  |  |
|  |  |  |  |  |  |
| **T_reg_ Cells** | 0.131±0.057 | 0.112±0.042 | 0.298±0.135 | 0.217±0.094 | 0.5616 |
|  |  |  |  |  |  |
| **CD8+ T Cells** | 1.537±0.719 | 1.076±0.346 | 1.608±0.529 | 0.986±0.490 | 0.7311 |
|  |  |  |  |  |  |
|  |  |  |  |  |  |
| **Natural Killer Cells** | 0.192±0.025 | 0.562±0.117 | 0.439±0.080 | 0.384±0.073 | 0.0997 |

Normal distribution of data was assessed using Shapiro-Wilk's normality test. For normally distributed data, one-way ANOVA was performed. For non-normally distributed data, Kruskal-Wallis test was performed using GraphPad Prism 9.3.1. *P*<0.05 was considered statistically significant. Data presented as mean ± SEM (x10^4^) cell counts unless otherwsie stated. n=4-11/group. Conventional dendritic cells type 1 and 2 (cDC1 and cDC2).

**Table S8.** Flow cytometric data, including immune cell counts and activation, of the peripheral blood from sham- and Ang II-challenged (0.75mg/kg body weight/day) wild-type (WT) and GPR68-deficient (Gpr68^-/-^) male mice.

| **Cell type** | **WT Sham** | **WT Ang II** | ***Gpr68*^-/-^ Sham** | ***Gpr68*^-/-^ Ang II** | ***P*-value** |
| --- | --- | --- | --- | --- | --- |
| **Immune Cells** | 62.42±23.33 | 45.74±18.51 | 71.05±23.70 | 58.30±24.95 | 0.8389 |
| **Macrophages** | 0.7182±0.2849 | 0.7556±0.3384 | 1.469±0.3937 | 0.7821±0.2921 | 0.4033 |
| **Inflammatory Monocytes** | 3.317±1.992 | 2.492±1.118 | 3.967±1.488 | 3.010±1.527 | 0.9010 |
| **Neutrophils** | 4.504±1.751 | 6.375±2.765 | 7.780±2.991 | 10.90±6.184 | 0.8108 |
| **Dendritic Cells** | 1.669±0.6447 | 1.001±0.4102 | 2.373±0.5091 | 1.872±0.7735 | 0.1973 |
|  |  |  |  |  |  |
| **cDC1** | 0.377±0.1793 | 0.2367±0.1108 | 0.4619±0.1344 | 0.2756±0.1268 | 0.2260 |
| **cDC2** | 1.194±0.4649 | 0.6952±0.2729 | 1.785±0.4379 | 1.461±0.6558 | 0.1803 |
| **B Cells** | 25.13±9.633 | 16.00±7.090 | 25.57±8.659 | 19.61±7.861 | 0.7897 |
|  |  |  |  |  |  |
|  |  |  |  |  |  |
| **γδ T Cells** | 0.9420±0.3736 | 0.5047±0.2339 | 0.8963±0.3596 | 0.7663±0.4023 | 0.7612 |
| **CD4+ T Cells** | 10.08±4.426 | 5.631±2.184 | 9.154±3.195 | 6.288±3.136 | 0.8077 |
|  |  |  |  |  |  |
|  |  |  |  |  |  |
| **T_reg_ Cells** | 0.5906±0.2542 | 0.2844±0.0997 | 0.4965±0.1637 | 0.4587±0.2179 | 0.8802 |
|  |  |  |  |  |  |
| **CD8+ T Cells** | 8.269±3.710 | 4.913±2.164 | 6.325±2.169 | 5.281±2.503 | 0.9670 |
|  |  |  |  |  |  |
|  |  |  |  |  |  |
| **Natural Killer Cells** | 0.4502±0.0975 | 0.6664±0.1130 | 0.6421±0.1557 | 0.7144±0.1221 | 0.5368 |

Normal distribution of data was assessed using Shapiro-Wilk's normality test. For normally distributed data, one-way ANOVA was performed. For non-normally distributed data, Kruskal-Wallis test was performed using GraphPad Prism 9.3.1. *P*<0.05 was considered statistically significant. Data presented as mean ± SEM (x10^4^) cell counts unless otherwsie stated. n=4-11/group. Conventional dendritic cells type 1 and 2 (cDC1 and cDC2).

**Table S9.** Flow cytometric data of the spleen from sham- and Ang II-challenged (0.75mg/kg body weight/day) wild-type (WT) and GPR68-deficient (Gpr68^-/-^) male mice.

| **Cell type** | **WT Sham** | **WT Ang II** | ***Gpr68*^-/-^ Sham** | ***Gpr68*^-/-^ Ang II** | ***P*-value** |
| --- | --- | --- | --- | --- | --- |
| **Immune Cells** | 34.51±5.811 | 33.42±3.361 | 31.29±4.952 | 27.83±5.139 | 0.7734 |
| **Macrophages (x10^3^)** | 0.2423±6.132e-002 | 0.3307±0.1161 | 0.0001960±2.222e-005 | 0.0002585±0.0001272 | 0.6478 |
| **Inflammatory Monocytes** | 0.0006129±0.0001899 | 0.0003712±6.132e-005 | 0.0005035±0.0001379 | 0.0007058±0.0002639 | 0.6517 |
| **Neutrophils (x10^3^)** | 1.532±0.4221 | 1.215±0.2807 | 0.001502±0.0003471 | 0.002071±0.0006219 | 0.6766 |
| **Dendritic Cells** | 0.002062±0.0004093 | 0.001861±0.0002389 | 0.001707±0.0002468 | 0.001701±0.0003147 | 0.8668 |
|  |  |  |  |  |  |
| **B Cells** | 0.01805±0.005372 | 0.01607±0.002479 | 0.01813± 0.003298 | 0.02355±0.007010 | 0.7301 |
|  |  |  |  |  |  |
|  |  |  |  |  |  |
| **γδ T Cells** | 0.0003015±5.710e-005 | 0.0002815±3.608e-005 | 0.0003539±8.427e-005 | 0.0005646±0.0002059 | 0.3967 |
| **CD4+ T Cells** | 0.006136±0.001378 | 0.006179±0.0009051 | 0.007781±0.001603 | 0.01185±0.005125 | 0.5353 |
|  |  |  |  |  |  |
|  |  |  |  |  |  |
| **T_reg_ Cells** | 0.0008188±0.0002627 | 0.0008449±0.0001347 | 0.001041±0.0002179 | 0.001452±0.0005681 | 0.7436 |
|  |  |  |  |  |  |
| **CD8+ T Cells** | 0.004695±0.001093 | 0.004527±0.0006848 | 0.005577±0.001264 | 0.008146±0.003279 | 0.8229 |
|  |  |  |  |  |  |
|  |  |  |  |  |  |
| **Natural Killer Cells** | 0.001395±0.0005072 | 0.001350±0.0002643 | 0.002212±0.0008178 | 0.002910±0.001320 | 0.7121 |

Normal distribution of data was assessed using Shapiro-Wilk's normality test. For normally distributed data, one-way ANOVA was performed. For non-normally distributed data, Kruskal-Wallis test was performed using GraphPad Prism 9.3.1. *P*<0.05 was considered statistically significant. Data presented as mean ± SEM (x10^6^) cell counts unless otherwsie stated. n=4-11/group. Mean fluorescence intensity (MFI).

**Table S10.** Flow cytometric data of the aorta from Ang II-challenged (0.75mg/kg body weight/day) wild-type (WT) and GPR68-deficient (*Gpr68*^-/-^) male mice fed either a control or high- fibre diet.

| **Cell type** | **WT Control Diet** | **WT High Fibre Diet** | ***Gpr68*^-/-^ Control Diet** | ***Gpr68*^-/-^ High Fibre Diet** | ***P*-value** |
| --- | --- | --- | --- | --- | --- |
|  |  |  |  |  |  |
| **CD4+ T Cells** | 52.31±32.66 | 12.77±8.347 | 6.331±3.592 | 0.2466±0.0600 | 0.0770 |
| **Natural Killer Cells** | 6.318±3.913 | 0.9911±0.6542 | 0.0567±0.0346 | 0.0611±0.0260 | 0.0927 |

Normal distribution of data was assessed using Shapiro-Wilk's normality test. Kruskal-Wallis test was performed using GraphPad Prism 9.3.1. *P*<0.05 was considered statistically significant (bold). Data presented as mean ± SEM (x10^3^) cell counts unless otherwsie stated. n=5-12/group.

**Table S11.** Flow cytometric data, including immune cell counts and activation, of the kidney from Ang II-challenged (0.75mg/kg body weight/day) wild-type (WT) and GPR68-deficient (*Gpr68*^-/-^) male mice fed either a control or high- fibre diet.

| **Cell type** | **WT Control Diet** | **WT High Fibre Diet** | ***Gpr68*^-/-^ Control Diet** | ***Gpr68*^-/-^ High Fibre Diet** | ***P*-value** |
| --- | --- | --- | --- | --- | --- |
| **Inflammatory Monocytes** | 0.5918±0.2524 | 0.2166±0.0561 | 0.3893±0.1246 | 0.2411±0.0824 | 0.5782 |
|  |  |  |  |  |  |
| **Dendritic Cells** | 25.94±11.13 | 6.564±1.180 | 13.02±5.403 | 7.535±2.055 | 0.1082 |
|  |  |  |  |  |  |
|  |  |  |  |  |  |
|  |  |  |  |  |  |
| **γδ T Cells** | 28.81±12.02 | 7.681±1.322 | 15.02±6.507 | 8.747±3.140 | 0.1773 |
| **CD4+ T Cells** | 2.400±1.052 | 0.6160±0.1257 | 1.808±0.7413 | 1.078±0.5604 | 0.1496 |
|  |  |  |  |  |  |
|  |  |  |  |  |  |
| **T_reg_ Cells** | 0.1118±0.0421 | 0.0419±0.0081 | 0.2166±0.0941 | 0.0791±0.0392 | 0.0421 |
|  |  |  |  |  |  |
|  |  |  |  |  |  |
|  |  |  |  |  |  |
| **Natural Killer Cells** | 0.5617±0.1174 | 0.5030±0.1047 | 0.3837±0.0728 | 0.5298±0.2281 | 0.9045 |

Normal distribution of data was assessed using Shapiro-Wilk's normality test. Kruskal-Wallis test was performed using GraphPad Prism 9.3.1. *P*<0.05 was considered statistically significant (bold). Data presented as mean ± SEM (x10^4^) cell counts unless otherwsie stated. n=5-12/group.

**Table S12.** Flow cytometric data, including immune cell counts and activation, of the peripheral blood from Ang II-challenged (0.75mg/kg body weight/day) wild-type (WT) and GPR68-deficient (Gpr68^-/-^) male mice fed either a control or high- fibre diet.

| **Cell type** | **WT Control Diet** | **WT High Fibre Diet** | ***Gpr68*^-/-^ Control Diet** | ***Gpr68*^-/-^ High Fibre Diet** | ***P*-value** |
| --- | --- | --- | --- | --- | --- |
| **Immune Cells** | 45.74±18.51 | 10.89±1.394 | 58.30±24.95 | 20.68±8.447 | 0.3068 |
| **Macrophages** | 0.7556±0.3384 | 0.7311±0.4481 | 0.7821±0.2921 | 0.1317±0.0256 | 0.8531 |
| **Inflammatory Monocytes** | 2.492±1.118 | 0.4675±0.0861 | 3.010±1.527 | 2.014±1.231 | 0.2487 |
| **Neutrophils** | 6.375±2.765 | 1.192±0.2134 | 10.90±6.184 | 4.958±2.233 | 0.0274 |
| **Dendritic Cells** | 1.001±0.4102 | 0.9755±0.5909 | 1.872±0.7735 | 0.6009±0.3991 | 0.2922 |
|  |  |  |  |  |  |
| **cDC1** | 0.2367±0.1108 | 0.0468±0.0162 | 0.2756±0.1268 | 0.0516±0.0175 | 0.1451 |
| **cDC2** | 0.6952±0.2729 | 0.8977±0.5584 | 1.461±0.6558 | 0.5352±0.3785 | 0.4392 |
| **B Cells** | 16.00±7.090 | 3.774±0.6577 | 19.61±7.861 | 5.369±1.193 | 0.1253 |
|  |  |  |  |  |  |
|  |  |  |  |  |  |
| **γδ T Cells** | 0.5047±0.2339 | 0.0869±0.0116 | 0.7663±0.4023 | 0.1556±0.0496 | 0.3467 |
| **CD4+ T Cells** | 5.631±2.184 | 1.171±0.2315 | 6.288±3.136 | 1.708±0.7101 | 0.4775 |
|  |  |  |  |  |  |
|  |  |  |  |  |  |
| **T_reg_ Cells** | 0.2844±0.0997 | 0.0661±0.0151 | 0.4587±0.2179 | 0.1166±0.0346 | 0.2504 |
|  |  |  |  |  |  |
| **CD8+ T Cells** | 4.913±2.164 | 1.026±0.1940 | 5.281±2.503 | 1.387±0.4750 | 0.6970 |
|  |  |  |  |  |  |
|  |  |  |  |  |  |
| **Natural Killer Cells** | 0.6664±0.1130 | 0.7379±0.1036 | 0.7144±0.1221 | 1.240±0.5427 | 0.9726 |

Normal distribution of data was assessed using Shapiro-Wilk's normality test. For normally distributed data, one-way ANOVA was performed. For non-normally distributed data, Kruskal-Wallis test was performed using GraphPad Prism 9.3.1. *P*<0.05 was considered statistically significant (bold). Data presented as mean ± SEM (x10^4^)cell counts unless otherwsie stated. n=5-12/group. Conventional dendritic cells type 1 and 2 (cDC1 and cDC2).

**Table S13.** Flow cytometric data, including immune cell counts and activation, of the spleen from Ang II-challenged (0.75mg/kg body weight/day) wild-type (WT) and GPR68-deficient (Gpr68^-/-^) male mice fed either a control or high- fibre diet.

| **Cell type** | **WT Control Diet** | **WT High Fibre Diet** | ***Gpr68*^-/-^ Control Diet** | ***Gpr68*^-/-^ High Fibre Diet** | ***P*-value** |
| --- | --- | --- | --- | --- | --- |
| **Immune Cells** | 33.42±3.361 | 31.33±3.844 | 27.83±5.139 | 40.54±11.40 | 0.7965 |
| **Macrophages** | 0.0003307±0.0001161 | 0.0001942±3.001e-005 | 0.0002644±0.0001468 | 9.616e-005±1.922e-005 | 0.0584 |
| **Inflammatory Monocytes** | 0.0003712±6.132e-005 | 0.0005615±0.0001594 | 0.0007058±0.0002639 | 0.0005655±0.0001019 | 0.3733 |
| **Neutrophils** | 0.001215±0.0002807 | 0.001680±0.0005278 | 0.002071±0.0006219 | 0.002143±0.0004590 | 0.2313 |
| **Dendritic Cells** | 0.001861±0.0002389 | 0.002065±0.0003913 | 0.001701±0.0003147 | 0.001344±0.0002135 | 0.4900 |
|  |  |  |  |  |  |
| **B Cells** | 0.01607±0.002479 | 0.01555±0.001683 | 0.02355±0.007010 | 0.01641±0.003821 | 0.8241 |
|  |  |  |  |  |  |
|  |  |  |  |  |  |
| **γδ T Cells** | 0.0002815±3.608e-005 | 0.0003485±6.122e-005 | 0.0005646±0.0002059 | 0.0003625±8.491e-005 | 0.4795 |
| **CD4+ T Cells** | 0.006179±0.0009051 | 0.007438±0.001178 | 0.01185±0.005125 | 0.007166±0.002230 | 0.6551 |
|  |  |  |  |  |  |
|  |  |  |  |  |  |
| **T_reg_ Cells** | 0.0008449±0.0001347 | 0.0008720±0.0001811 | 0.001452±0.0005681 | 0.0009624±0.0002593 | 0.9339 |
|  |  |  |  |  |  |
| **CD8+ T Cells** | 0.004527±0.0006848 | 0.005898±0.001143 | 0.008146±0.003279 | 0.005200±0.001517 | 0.7176 |
|  |  |  |  |  |  |
|  |  |  |  |  |  |
| **Natural Killer Cells** | 0.001350±0.0002643 | 0.001884±0.0005294 | 0.002910±0.001320 | 0.001306±0.0003151 | 0.5853 |

Normal distribution of data was assessed using Shapiro-Wilk's normality test. For normally distributed data, one-way ANOVA was performed. For non-normally distributed data, Kruskal-Wallis test was performed using GraphPad Prism 9.3.1. *P*<0.05 was considered statistically significant. Data presented as mean ± SEM (x10^6^) cell counts unless therwsie stated. n=5-12/group.

**Online supplementary figures**

**
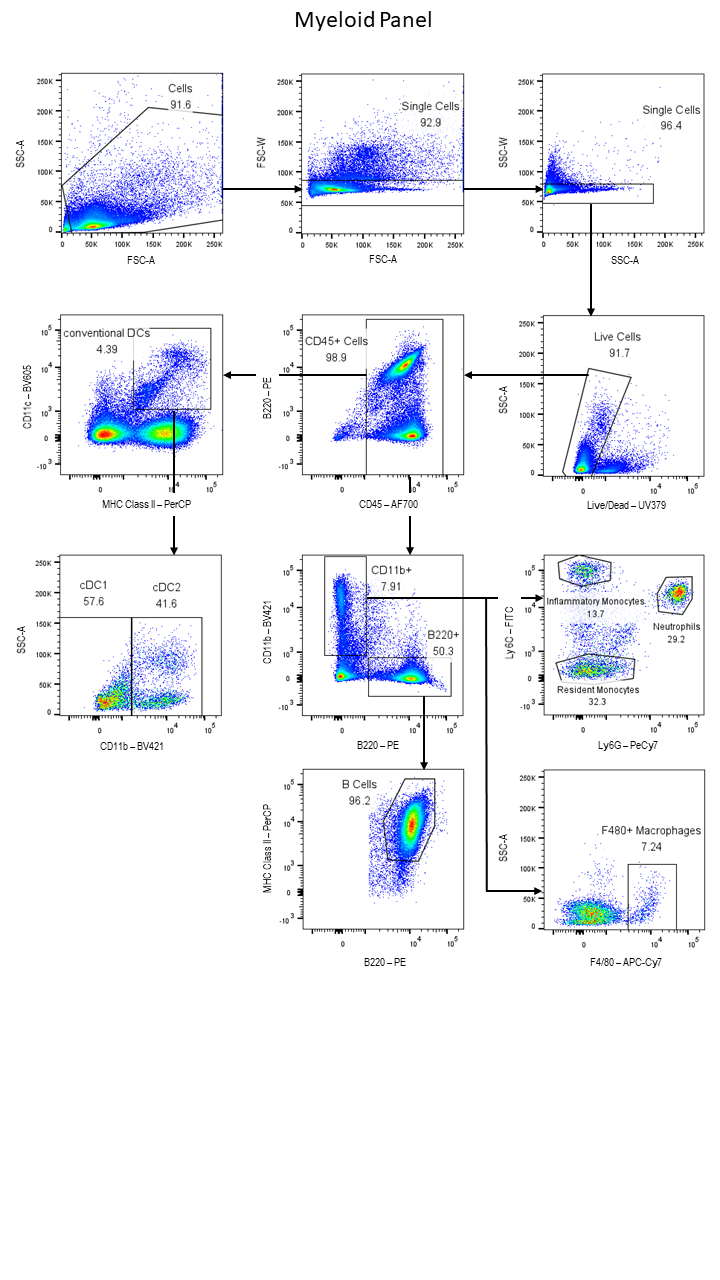
**

**Figure S1.** Flow cytometric gating strategy for myeloid cell populations and B cells in the kidney, spleen and peripheral blood. Type 1 and 2 conventional dendritic cells (cDC1 and cDC2, respectively) gating was only applied to non-lymphoid tissues including the kidney and peripheral blood, and excluded in spleen gating analysis. Data was analysed using FlowJo v10.10.0.


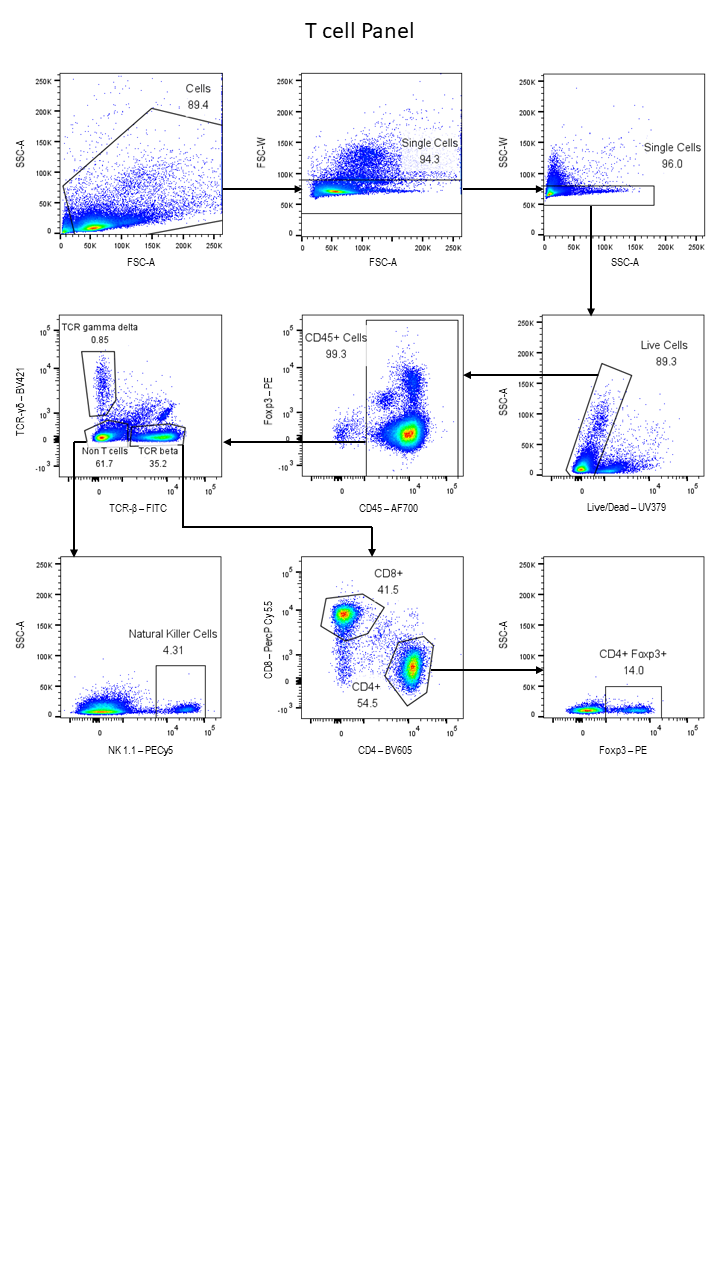
**Figure S2.** Flow cytometric gating strategy for T-cell populations in the kidney, spleen and peripheral blood. Data was analysed using FlowJo v10.10.0.


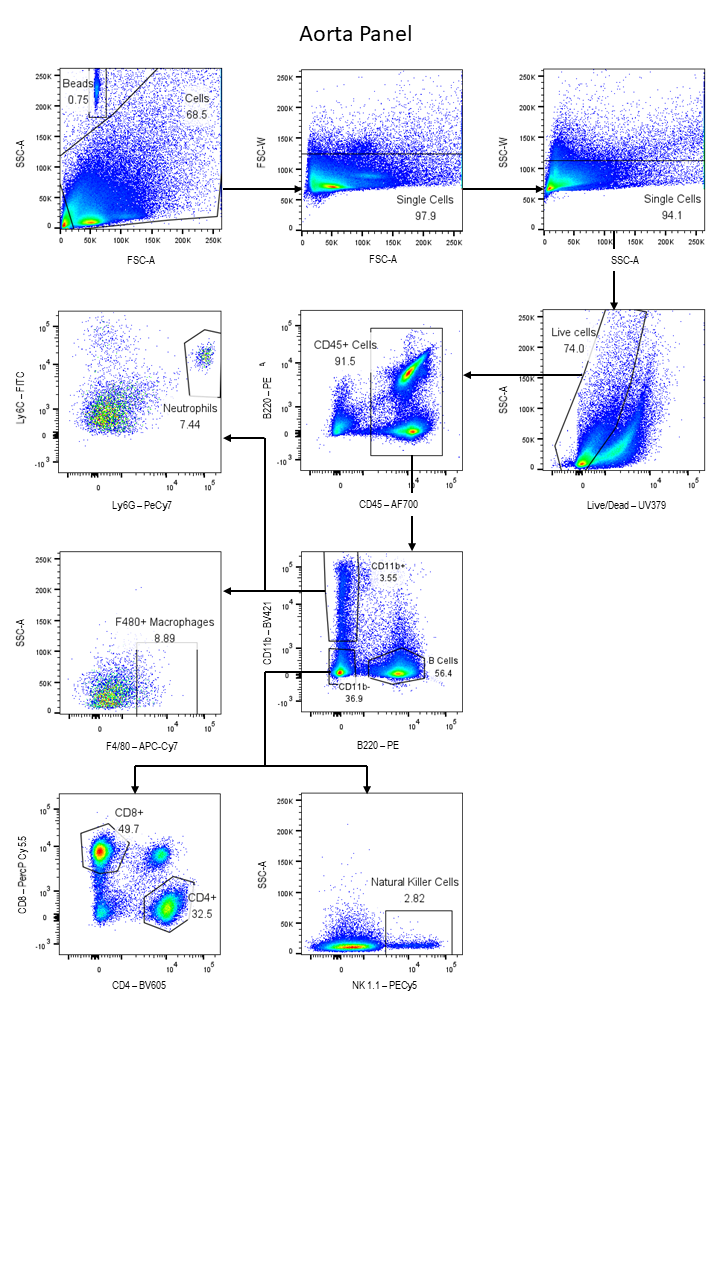


**Figure S3.** Flow cytometric gating strategy for myeloid, T-cell populations and B cells in the thoracic aorta. Data was analysed using FlowJo v10.10.0.


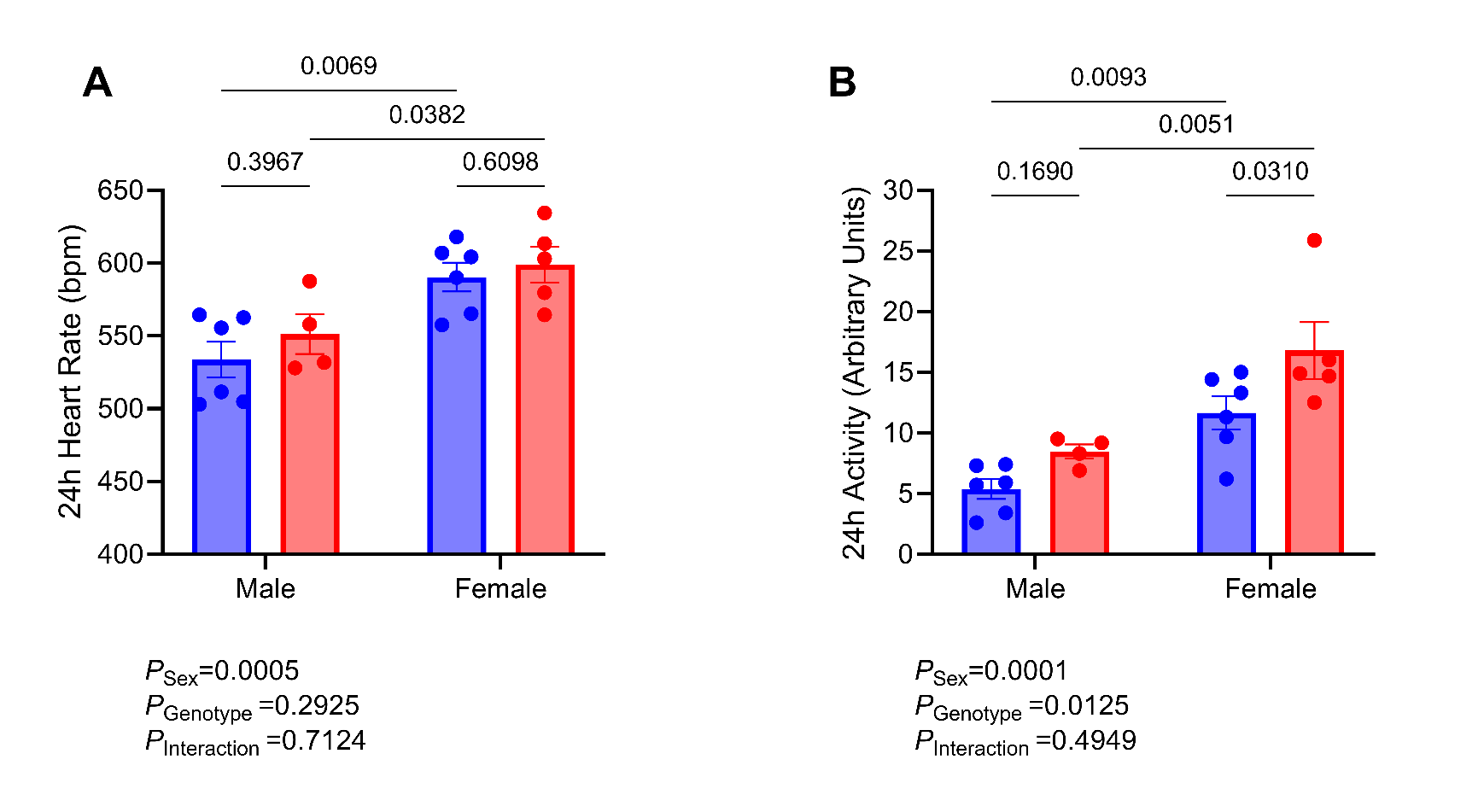


**Figure S4. 24-hour telemetry parameters of untreated wild-type (WT) and *Gpr68^-/-^* mice.** (A) 24-hour heart rate and (B) 24-hour activity of ten-to-twelve-week-old male and female WT and *Gpr68^-/-^* mice. Each data point represents an individual sample. Normal distribution of data was assessed using Shapiro-Wilk's normality test. Two-way ANOVA with Benjamini and Hochberg's false discovery rate adjustment for multiple comparisons was performed for normally distributed data. Data presented as mean ± SEM. n=4-11/group.


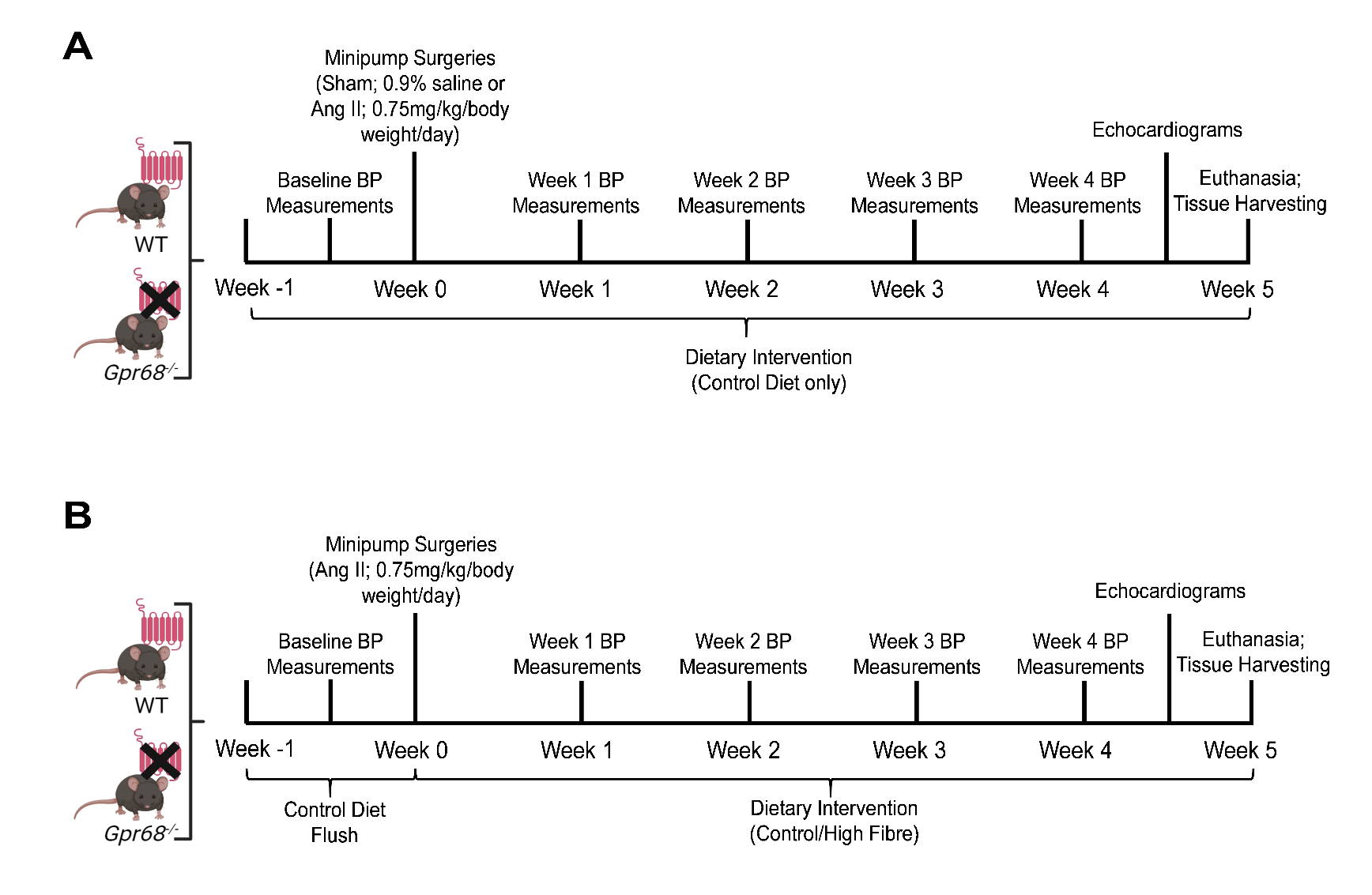
**Figure S5. Experimental designs** of (A) six-to-eight-week-old male and female WT and *Gpr68^-/-^* mice undergoing minipump implantations containing 0.9% sodium chloride (sham) or Angiotensin II (Ang II; 0.75 mg/kg body weight/day) or (B) six-to-eight-week-old male WT and Gpr68-/- mice undergoing minipump implantations containing Angiotensin II (Ang II; 0.75 mg/kg body weight/day) for four weeks. BP was measured weekly using a tail-cuff device. the day before euthanasia, mice underwent cardiac ultrasounds.


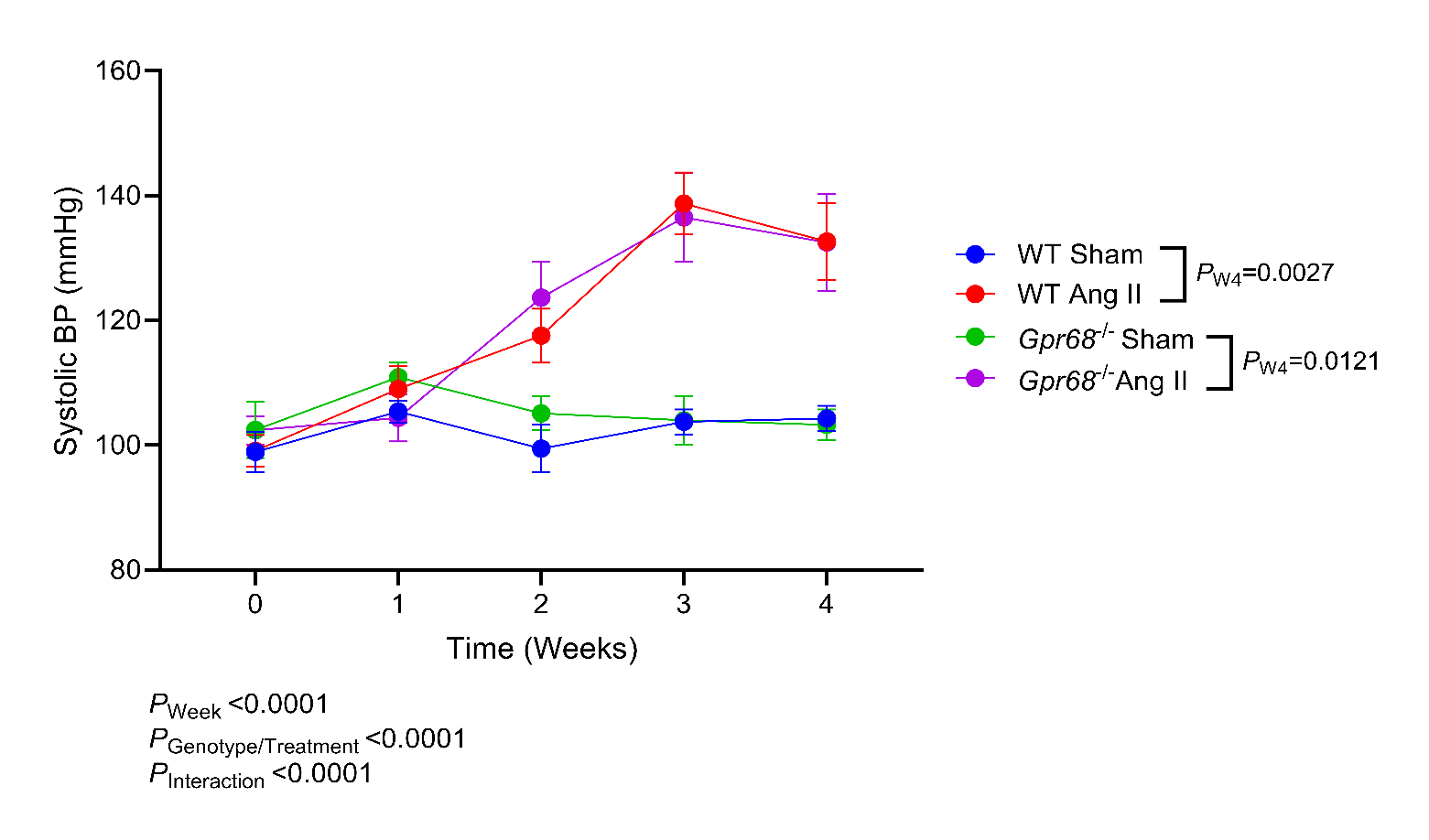


**Figure S6. Weekly systolic blood pressure (BP) of male wild-type (WT) and *Gpr68^-/-^* mice challenged with 0.9% sodium chloride (sham) or Angiotensin II (Ang II; 0.75 mg/kg body weight/day) while on a control diet.** Two-way ANOVA with Benjamini and Hochberg's false discovery rate adjustment for multiple comparisons was performed for normally distributed data. Data presented as mean ± SEM. n=8-11/group.

**
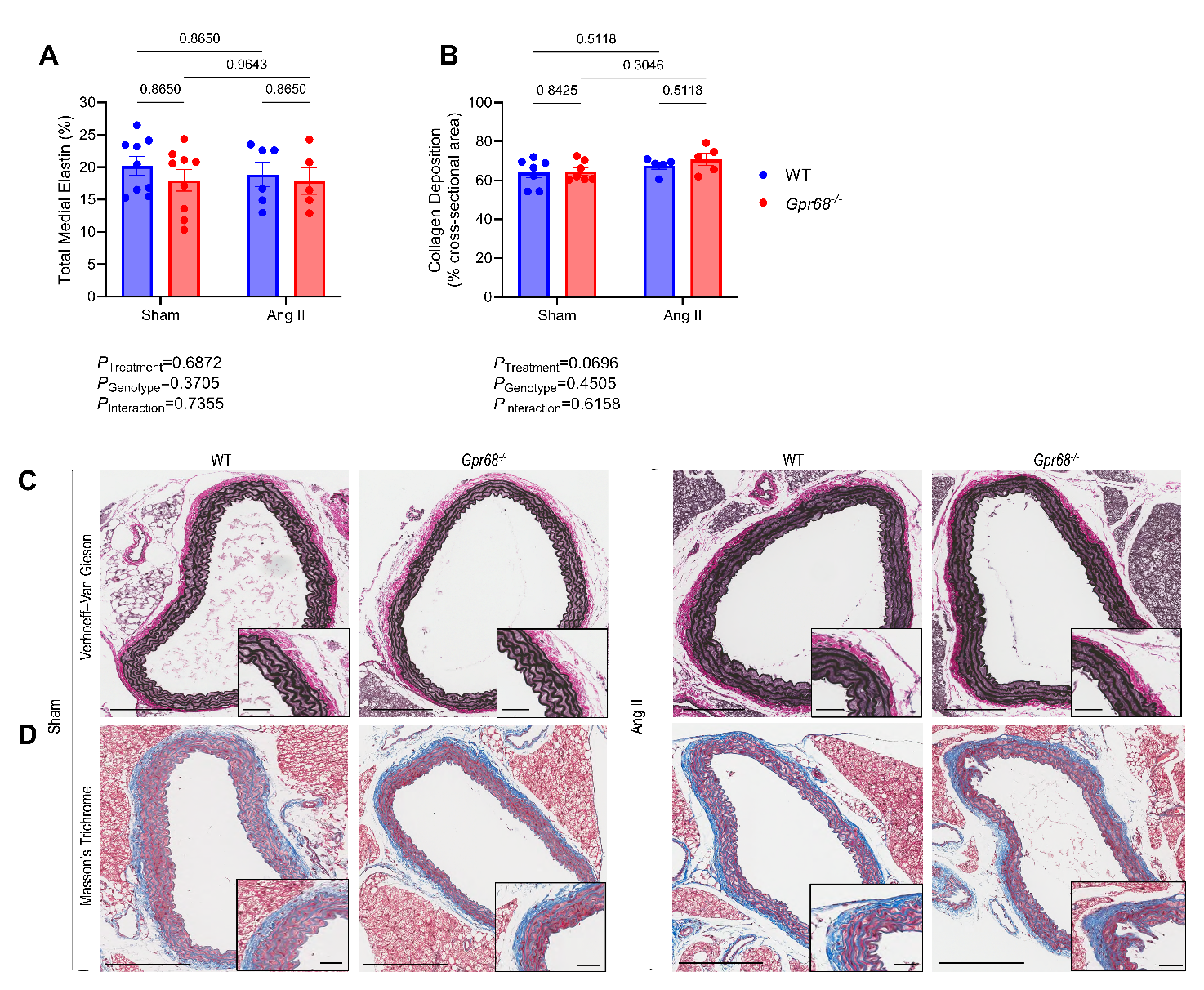
Figure S7. Arterial parameters of male wild-type (WT) and *Gpr68^-/-^* mice challenged with** **0.9% sodium chloride (sham) or Angiotensin II (Ang II) while on a control diet.** (A) Percentage of aortic total medial elastin content, (B) percentage of collagen deposition surrounding the wall of the aorta, (C) Verhoeff–Van Gieson-stained aortic sections showing elastin deposition and (D) Masson’s trichrome-stained aortic sections showing collagen deposition of ten-to-twelve-week-old male WT and *Gpr68^-/-^* mice challenged with either 0.9% sodium chloride (sham) or Angiotensin II (Ang II; 0.75 mg/kg body weight/day). For representative aorta sections, zoomed out image scale bar = 200μm, zoomed in image scale bar = 50μm. Each data point represents an individual sample. Normal distribution of data was assessed using Shapiro-Wilk's normality test. Two-way ANOVA with Benjamini and Hochberg's false discovery rate adjustment for multiple comparisons was performed for normally distributed data. Data presented as mean ± SEM. n=5-9/group.


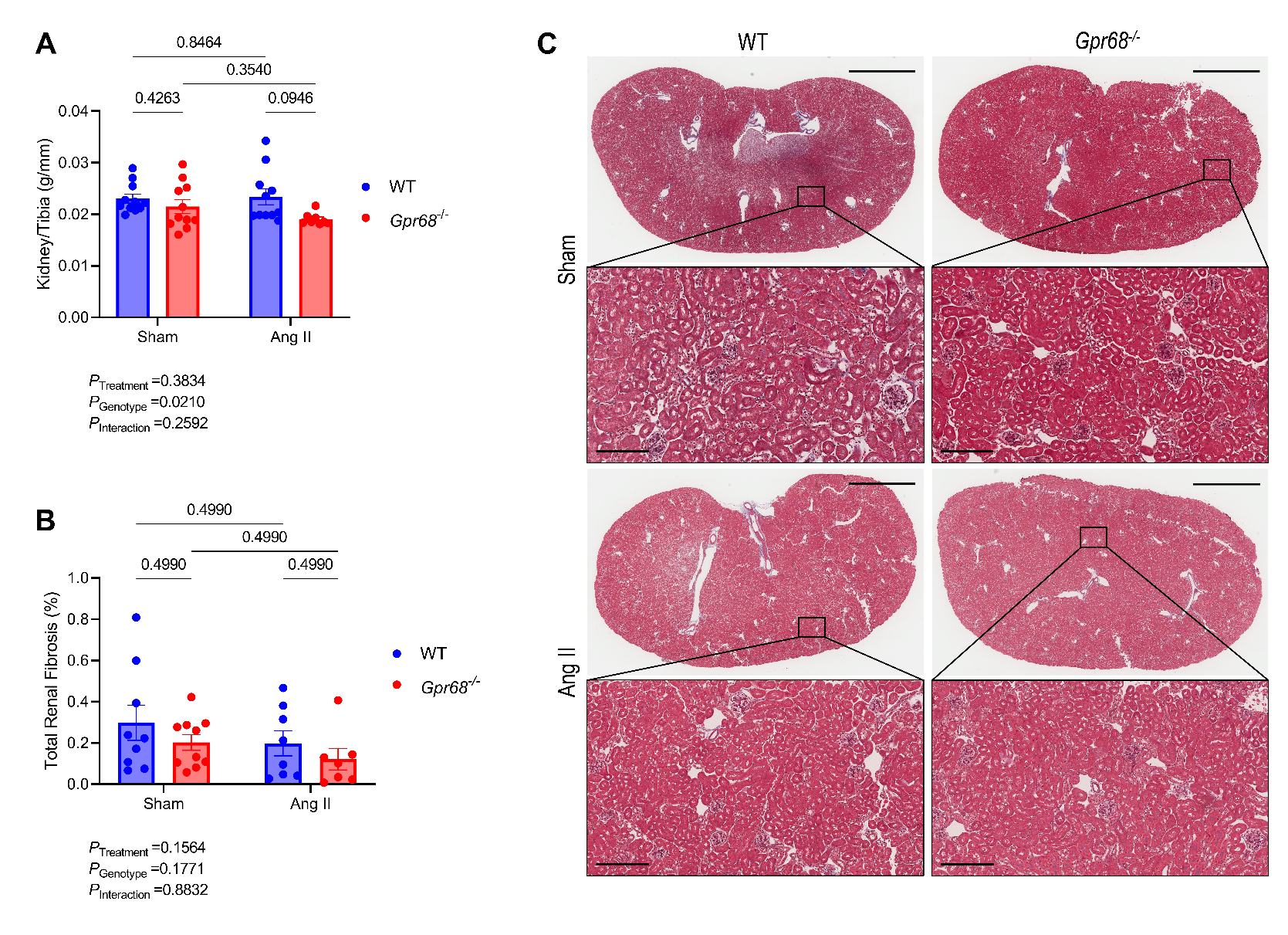
**Figure S8. Renal weight and fibrosis of male wild-type (WT) and *Gpr68^-/-^* mice challenged with 0.9% sodium chloride (sham) or Angiotensin II (Ang II) while on a control diet.** (A) Kidney weight to tibia length index, (B) percentage of total renal fibrosis (collagen deposition; blue) and (C) Masson’s trichrome-stained kidney sections of ten-to-twelve-week-old male WT and *Gpr68^-/-^* mice challenged with either 0.9% sodium chloride (sham) or Angiotensin II (Ang II; 0.75 mg/kg body weight/day). For representative kidney sections, upper panels scale bar = 2mm; for lower panels scale bar = 200μm. Each data point represents an individual sample. Normal distribution of data was assessed using Shapiro-Wilk's normality test. Two-way ANOVA with Benjamini and Hochberg's false discovery rate adjustment for multiple comparisons was performed for normally distributed data. Data presented as mean ± SEM. n=5-11/group.


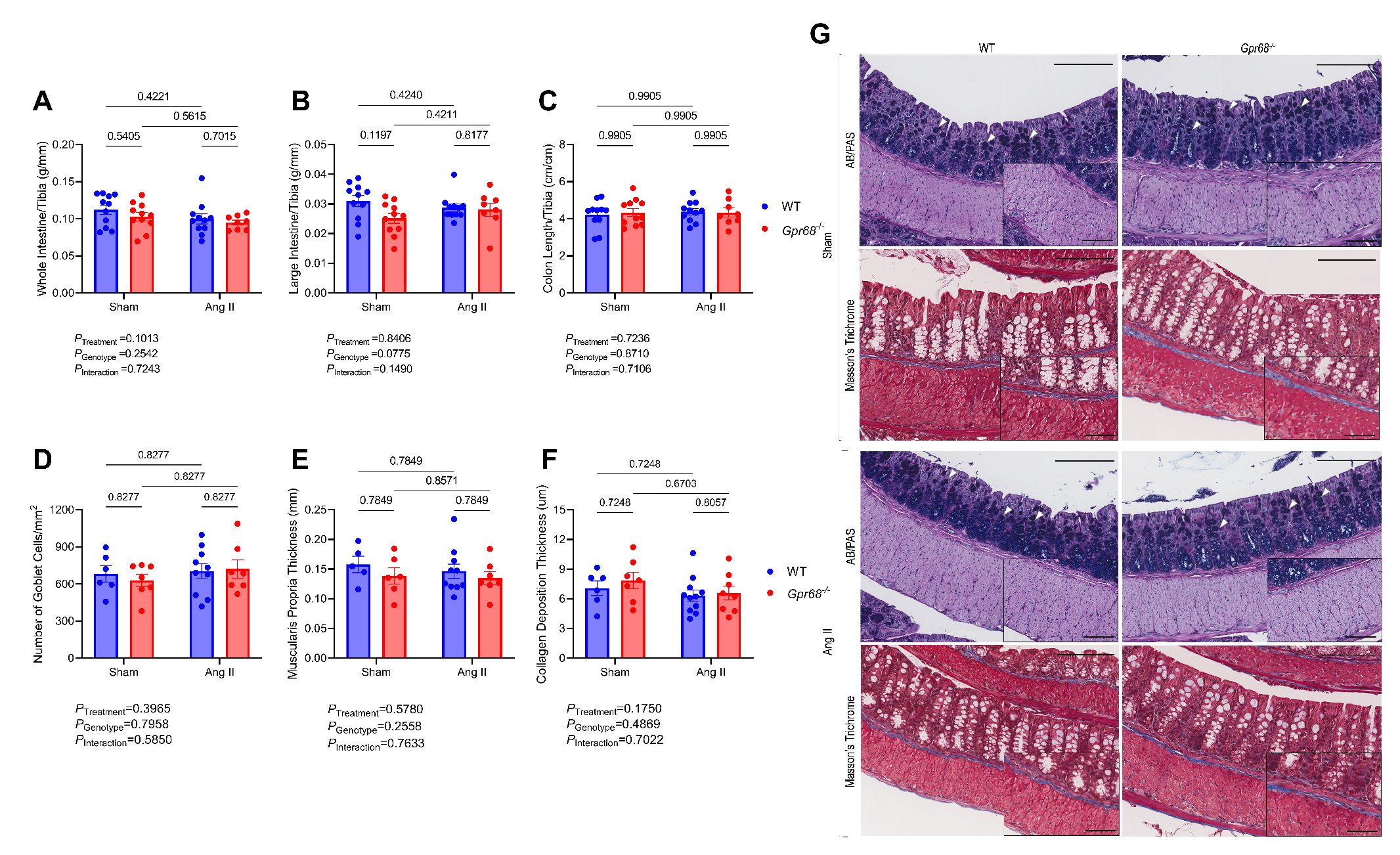
**Figure S9. Intestinal characterisation of male wild-type (WT) and *Gpr68^-/-^* mice challenged with 0.9% sodium chloride (sham) or Angiotensin II (Ang II) while on a control diet.** (A) Whole intestinal and (B) large intestinal weight to tibia length index, (C) colon length to tibia length index, (D) number of goblet cells (indicated by arrows), (E) thickness of muscularis propria layer, (F) collagen deposition thickness (blue) and (G) Masson’s trichrome- and Alcian blue/periodic acid–Schiff-stained colon sections of ten-to-twelve-week-old male WT and *Gpr68^-/-^* mice challenged with either 0.9% sodium chloride (sham) or Angiotensin II (Ang II; 0.75 mg/kg body weight/day). For representative colon sections, zoomed-out image scale bar = 200μm, zoomed-in image scale bar = 100μm. Each data point represents an individual sample. Normal distribution of data was assessed using Shapiro-Wilk's normality test. Two-way ANOVA with Benjamini and Hochberg's false discovery rate adjustment for multiple comparisons was performed for normally distributed data. Data presented as mean ± SEM. n=5-11/group.


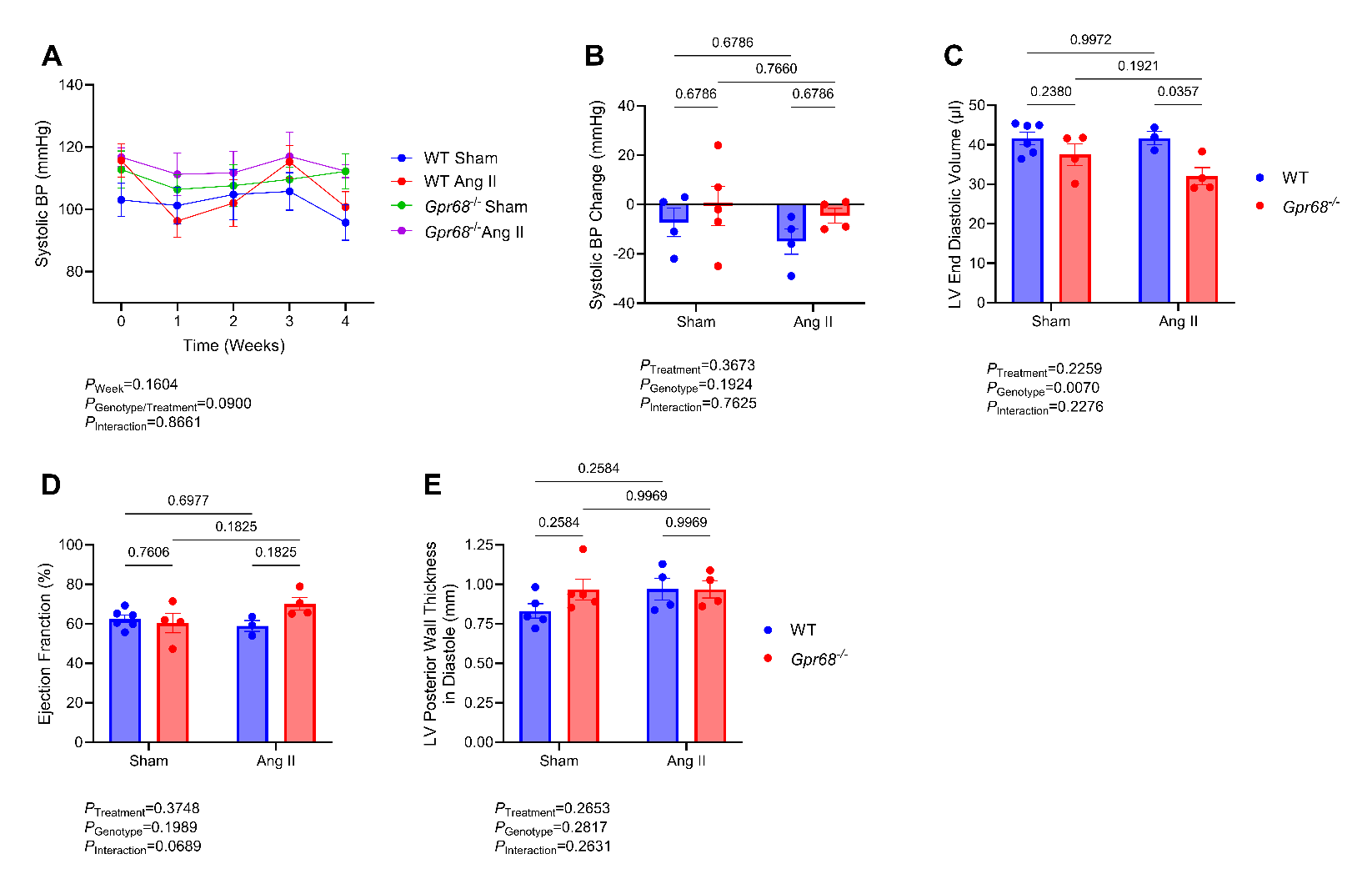
**Figure S10. Cardiovascular parameters of female wild-type (WT) and *Gpr68^-/-^* mice challenged with 0.9% sodium chloride (sham) or Angiotensin II (Ang II) while on a control diet.** (A) Systolic and (B) systolic BP change (the difference between baseline and week four systolic BP, (C) left ventricular (LV) end diastolic volume , (D) ejection fraction and (E)left ventricular (LV) posterior wall thickness in diastole of ten-to-twelve-week-old female WT and *Gpr68^-/-^* mice challenged with either 0.9% sodium chloride (sham) or Angiotensin II (Ang II; 0.75 mg/kg body weight/day). Each data point represents an individual sample. Normal distribution of data was assessed using Shapiro-Wilk's normality test. Two-way ANOVA with Benjamini and Hochberg's false discovery rate adjustment for multiple comparisons was performed for normally distributed data. Data presented as mean ± SEM. n=4-6/group.


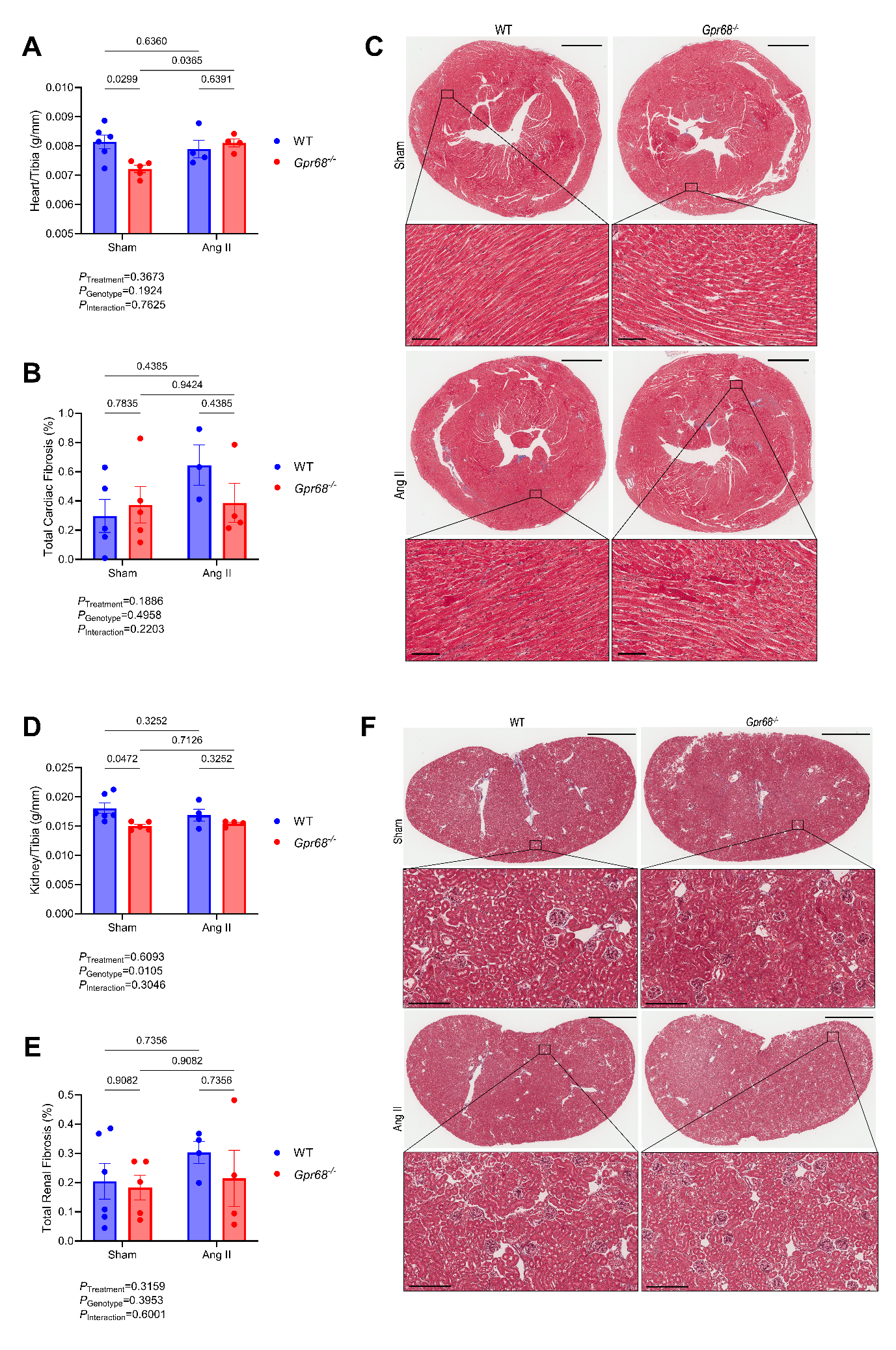
**Figure S11. Cardiac and renal fibrosis of female wild-type (WT) and *Gpr68^-/-^* mice challenged with 0.9% sodium chloride (sham) or Angiotensin II (Ang II) while on a control diet.** (A) Heart weight to tibia length index, (B) percentage of total cardiac fibrosis (collagen deposition; blue), (C) Masson’s trichrome-stained heart sections, (D) kidney weight to tibia length index, (E) percentage of total renal fibrosis (collagen deposition; blue) and (F) Masson’s trichrome-stained kidney sections of ten-to-twelve-week-old female WT and *Gpr68^-/-^* mice challenged with either 0.9% sodium chloride (sham) or Angiotensin II (Ang II; 0.75 mg/kg body weight/day). For representative heart sections, upper panels scale bar = 2mm; for lower panels scale bar = 100μm. For representative kidney sections, upper panels scale bar = 2mm; for lower panels scale bar = 200μm. Each data point represents an individual sample. Normal distribution of data was assessed using Shapiro-Wilk's normality test. Two-way ANOVA with Benjamini and Hochberg's false discovery rate adjustment for multiple comparisons was performed for normally distributed data. Data presented as mean ± SEM. n=4-6/group.


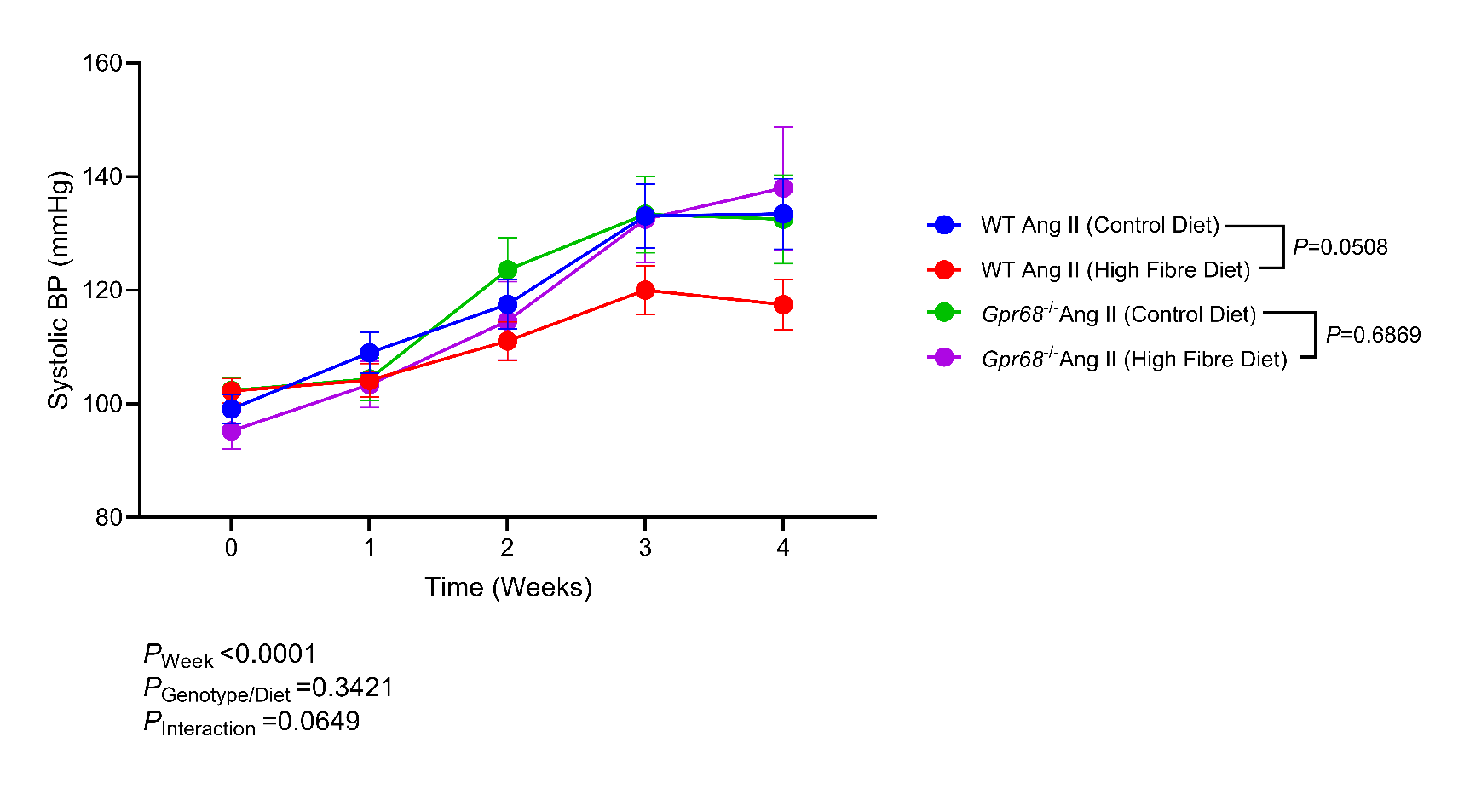
**Figure S12. Weekly systolic blood pressure (BP) of male wild-type (WT) and *Gpr68^-/-^* mice challenged with angiotensin II (Ang II; 0.75 mg/kg body weight/day) while on a control or high-fibre diet.** Two-way ANOVA with Benjamini and Hochberg's false discovery rate adjustment for multiple comparisons was performed for normally distributed data. Data presented as mean ± SEM. n=8-11/group.


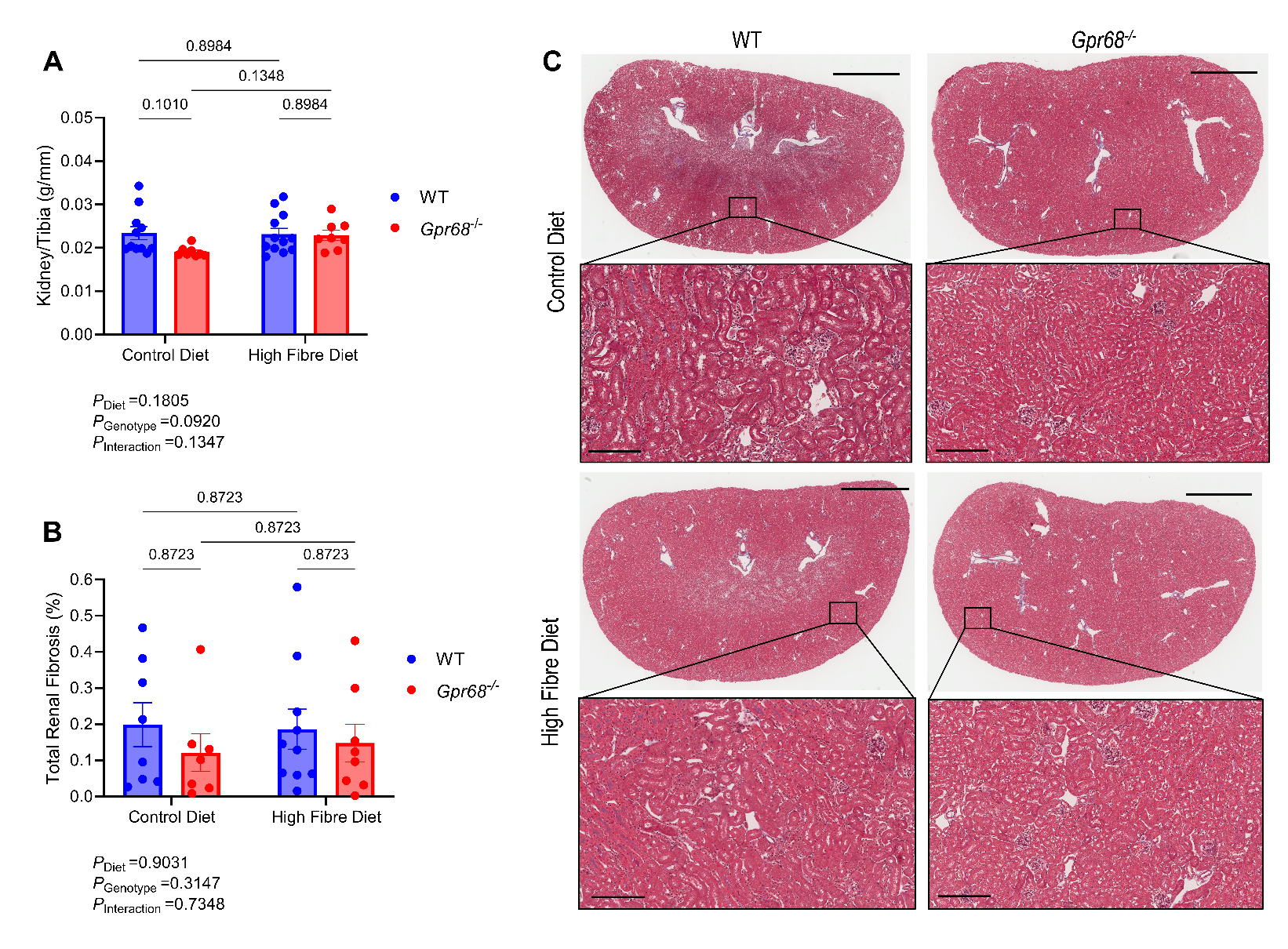
**Figure S13. Renal parameters of male wild-type (WT) and *Gpr68^-/-^* mice challenged with angiotensin II (Ang II) while on a control or high-fibre diet.** A) Kidney weight to tibia length index, (B) percentage of total renal fibrosis (collagen deposition; blue) and (C) Masson’s trichrome-stained kidney sections of ten-to-twelve-week-old male WT and *Gpr68^-/-^* mice challenged with Angiotensin II (Ang II; 0.75 mg/kg body weight/day). For representative kidney sections, upper panels scale bar = 2mm; for lower panels scale bar = 200μm. Each data point represents an individual sample. Normal distribution of data was assessed using Shapiro-Wilk's normality test. Two-way ANOVA with Benjamini and Hochberg's false discovery rate adjustment for multiple comparisons was performed for normally distributed data. Data presented as mean ± SEM. n=8-11/group.

**
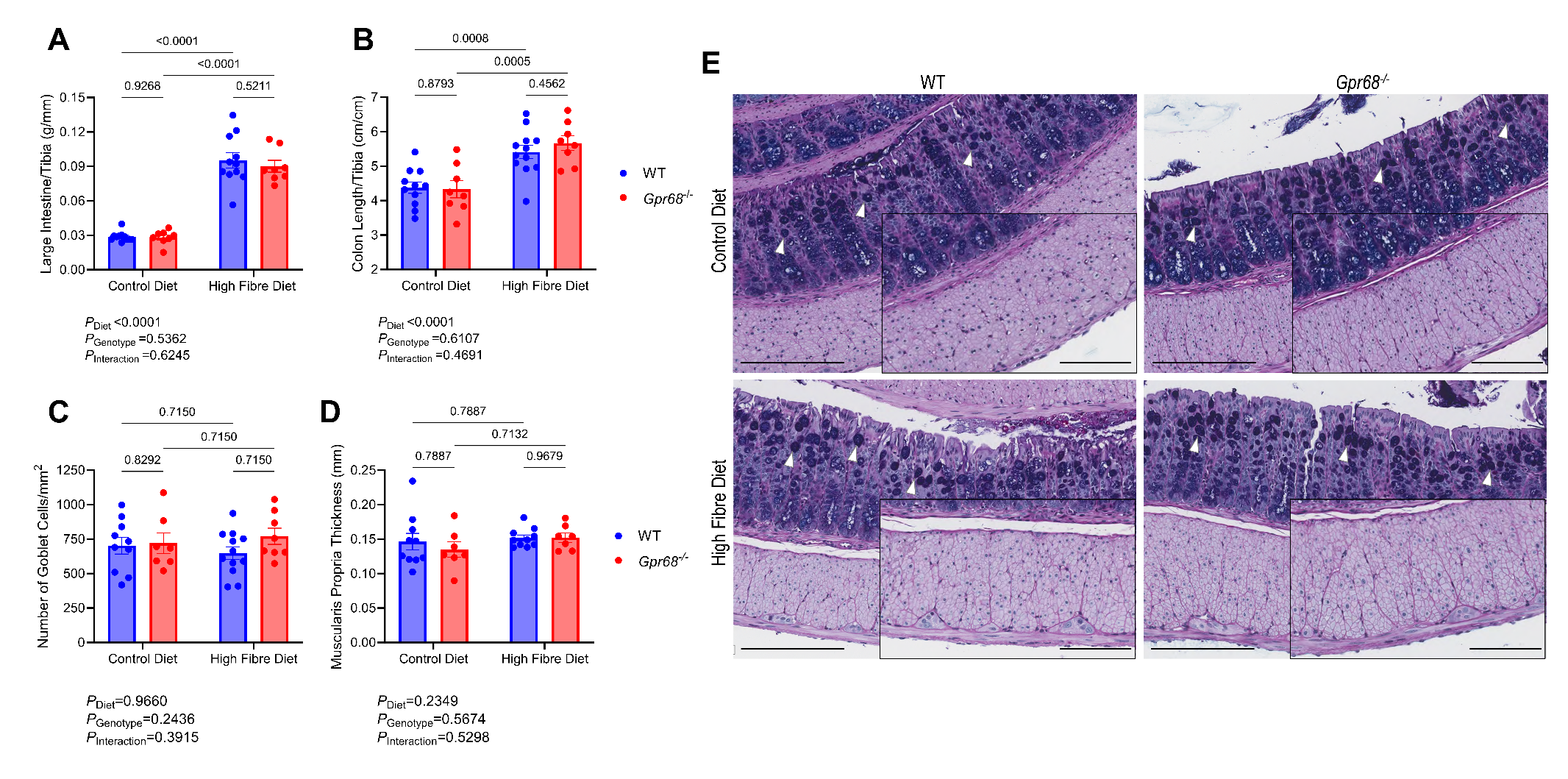
Figure S14. Intestinal parameters of wild-type (WT) and *Gpr68^-/-^* mice challenged with angiotensin II (Ang II) while on a control or high-fibre diet.** A) Normalized large intestinal weight and (B) colon length, (B) number of goblet cells (indicated by arrows), (D) thickness of muscularis propria layer and (E) Alcian blue/periodic acid–Schiff-stained colon sections. For representative colon sections, zoomed out image scale bar = 200μm, zoomed in image scale bar = 100μm. Each data point represents an individual sample. Normal distribution of data was assessed using Shapiro-Wilk's normality test. Two-way ANOVA with Benjamini and Hochberg's false discovery rate adjustment for multiple comparisons was performed for normally distributed data. Data presented as mean ± SEM. n=8-11/group.


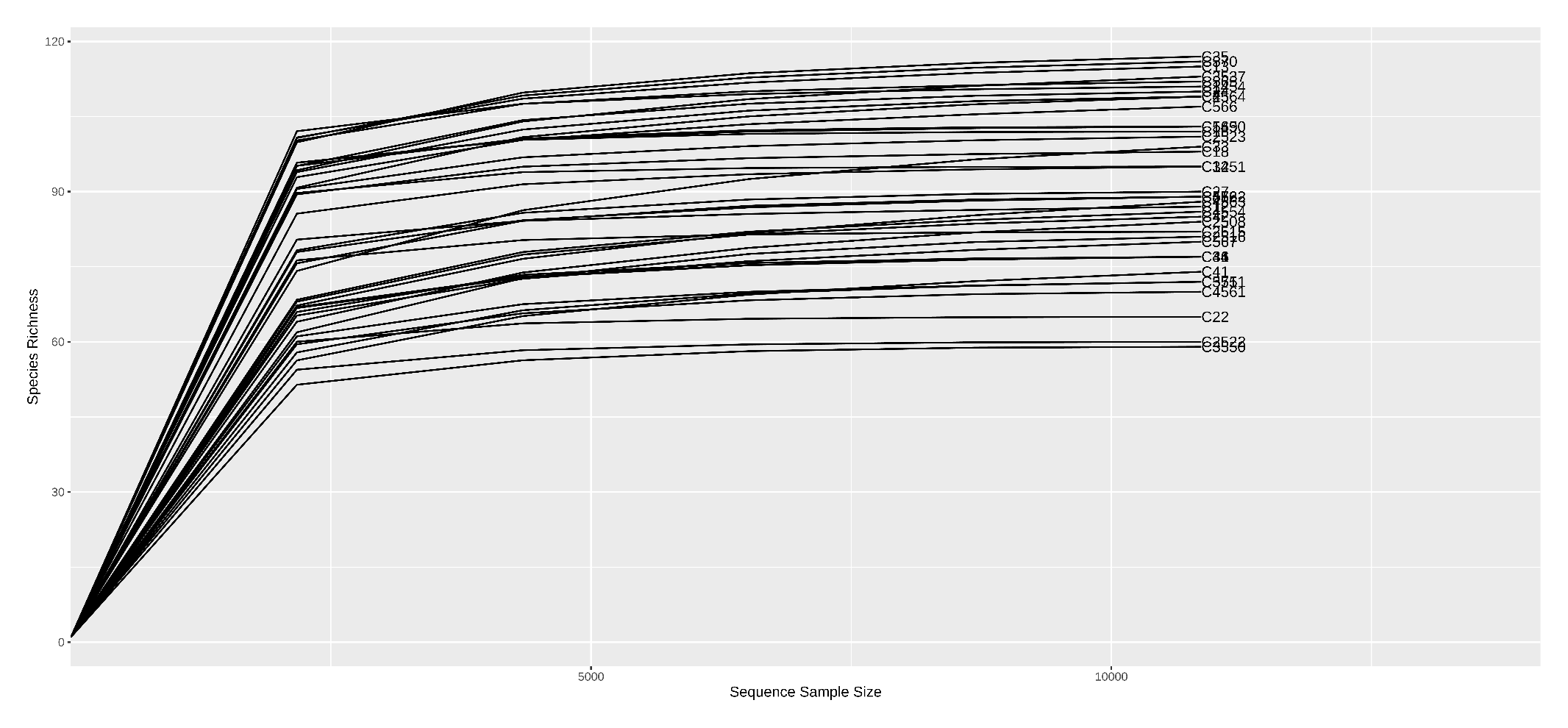


**Figure S15.**  **Rarefaction curve showing sample sequencing depth of hypertensive wildtype (WT) and GPR68-deficient (*Gpr68^-/-^*) male mice fed either a control or high-fibre diet**. n=39.


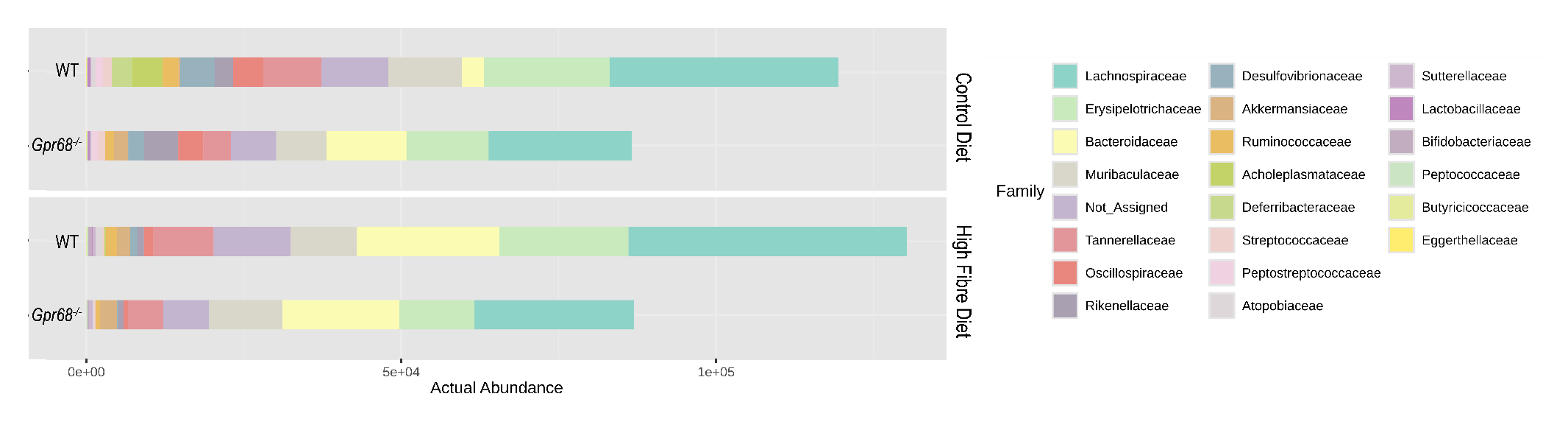


**Figure S16. Gut microbiome of wild-type (WT) and *Gpr68^-/-^* mice on a control or high-fibre diet.** Family-level abundance profiling of hypertensive wildtype (WT) and GPR68-deficient (Gpr68-/-) male mice fed either a control or high-fibre diet n=8-12/group.

**
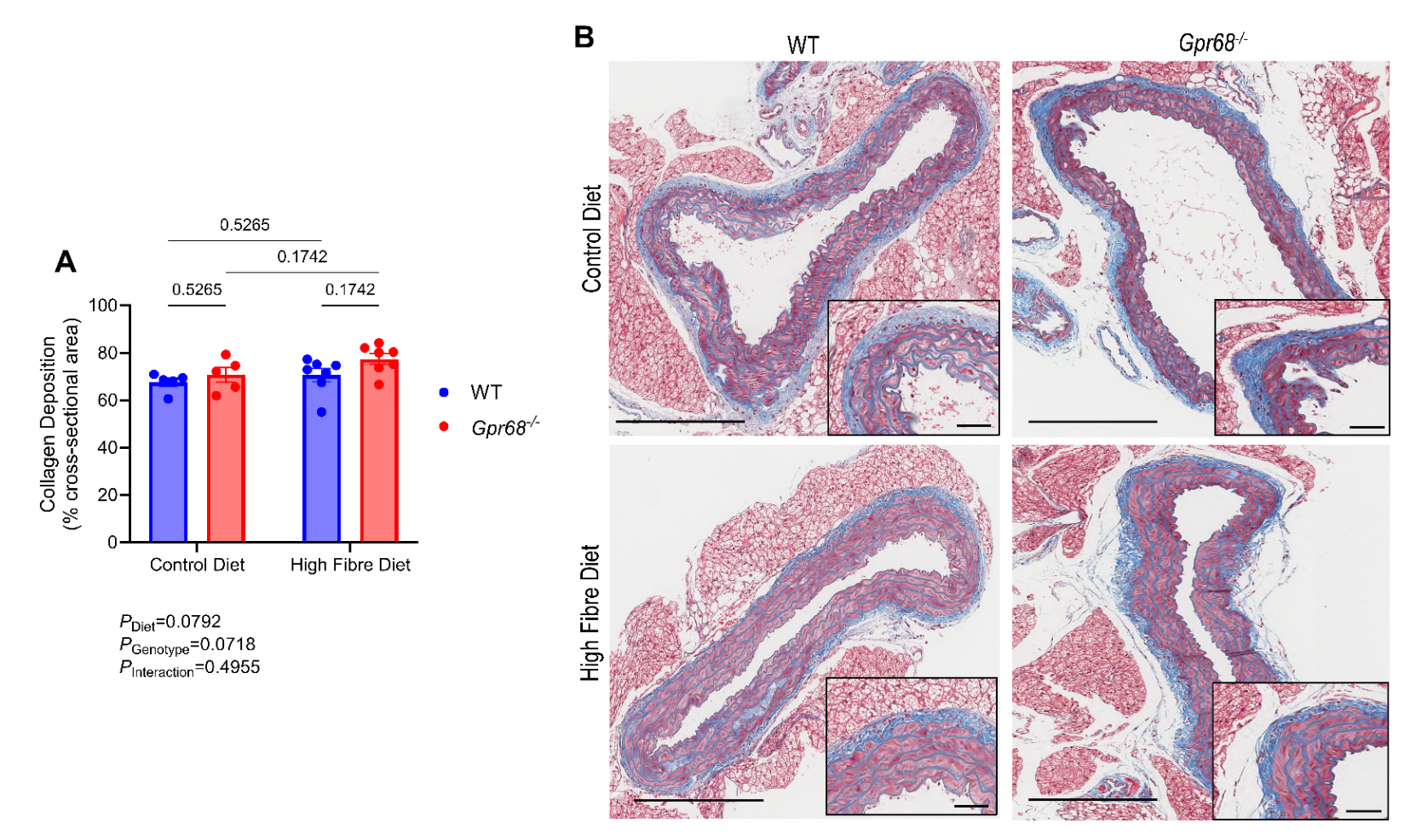
**

**Figure S17. Arterial parameters of male wild-type (WT) and *Gpr68^-/-^* mice challenged with angiotensin II (Ang II) while on a control or high-fibre diet.** (A) Percentage of collagen deposition surrounding the wall of the aorta and (B) Masson’s trichrome-stained aortic sections showing collagen deposition of ten-to-twelve-week-old male WT and *Gpr68^-/-^* mice challenged with Angiotensin II (Ang II; 0.75 mg/kg body weight/day). For representative aorta sections, zoomed out image scale bar = 200μm, zoomed in image scale bar = 50μm. Each data point represents an individual sample. Normal distribution of data was assessed using Shapiro-Wilk's normality test. Two-way ANOVA with Benjamini and Hochberg's false discovery rate adjustment for multiple comparisons was performed for normally distributed data. Data presented as mean ± SEM. n=5-7/group.
